# Supplementary figures and images for: Structural basis for the phase separation of the chromosome passenger complex
Source: eLife. 2024 Mar 8;13:e92709. doi: 10.7554/eLife.92709 (PMC10977965; doi:10.7554/eLife.92709)

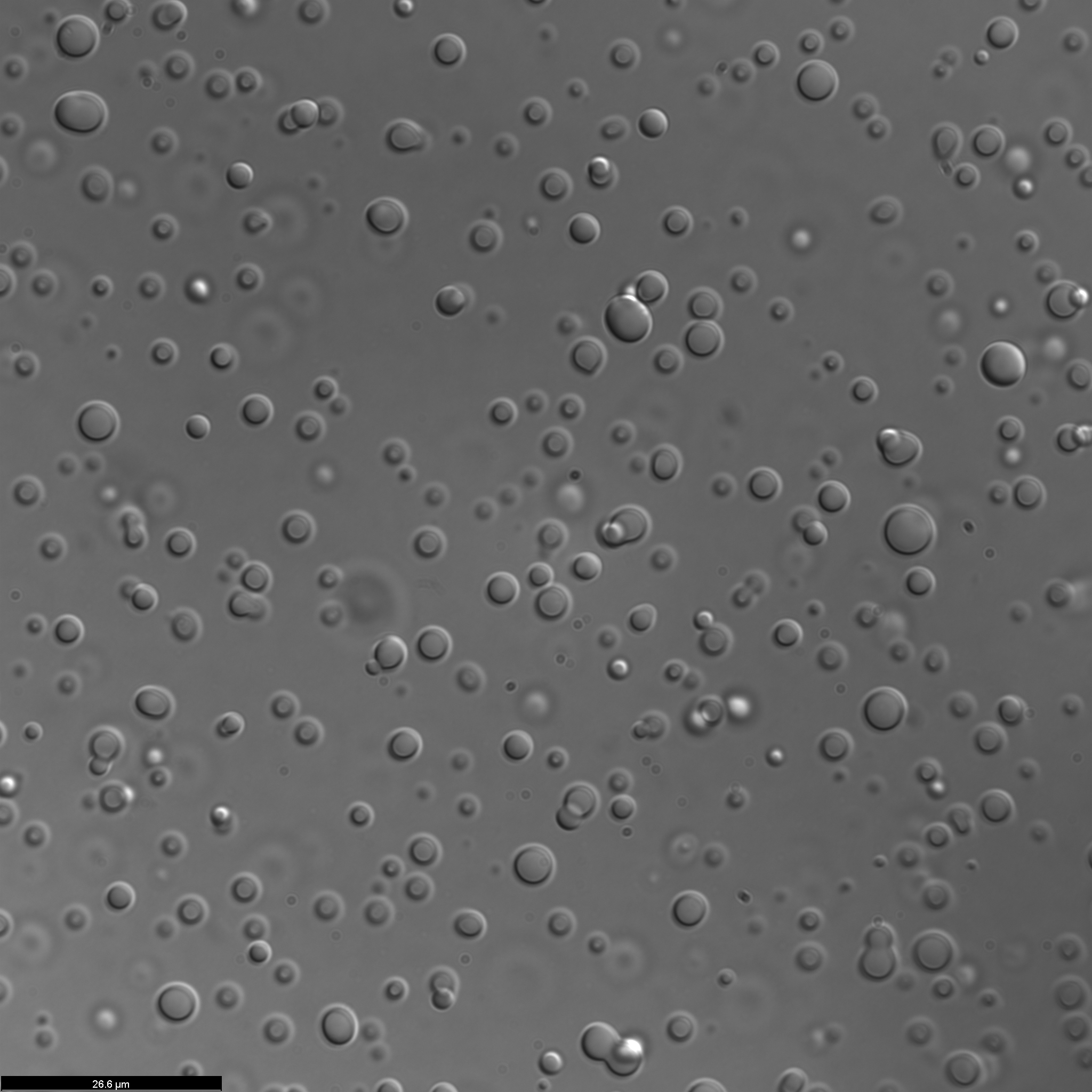

Supplement: Figure 1—source data 1. [file elife-92709-fig1-data1.zip › Figure 1 - Source Data/Figure 1 - source data 1 (Panel 1B)/20210507_ISB_TDP43_DropletAssay_WT Space 4 bottom_ch00.tif]

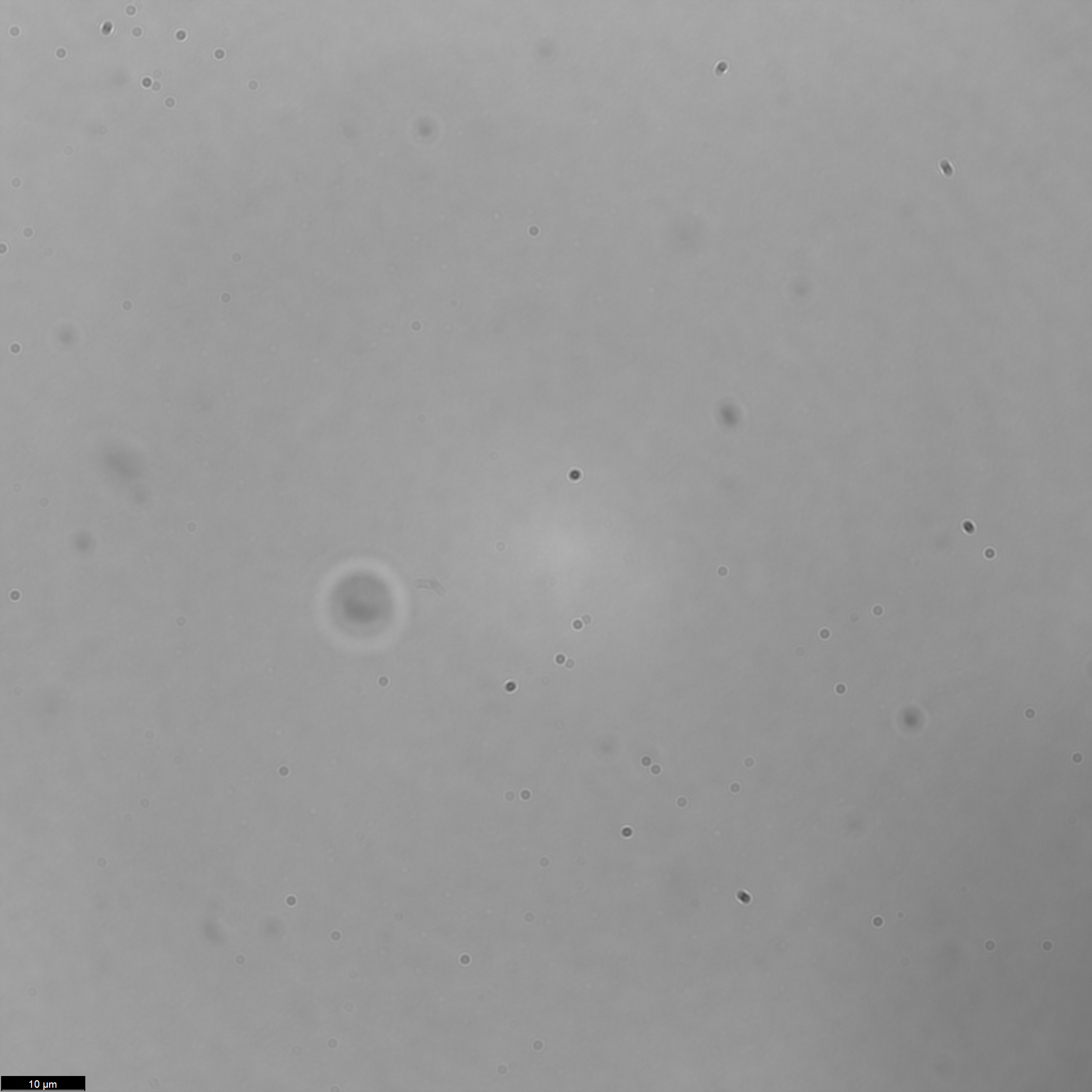

Supplement: Figure 1—source data 1. [file elife-92709-fig1-data1.zip › Figure 1 - Source Data/Figure 1 - source data 1 (Panel 1B)/20210826_WTISB_DropletAssay_Image018_ch00 - Blank.tif]

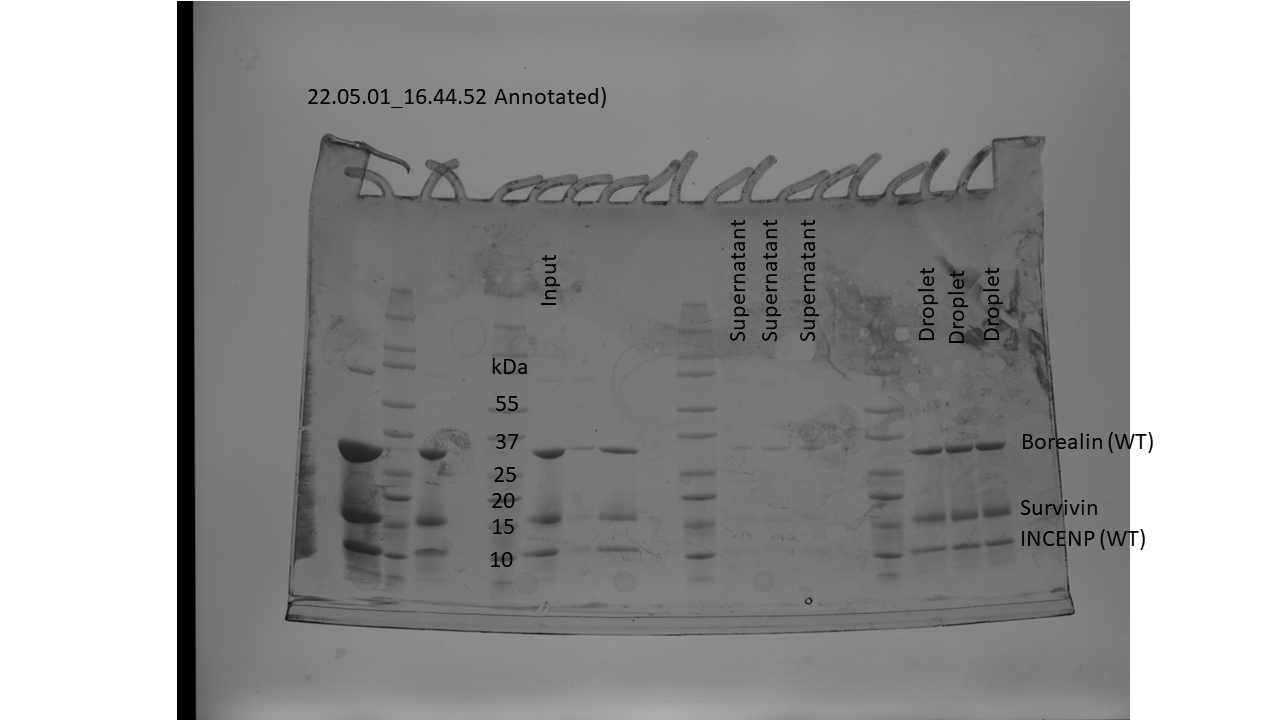

Supplement: Figure 1—source data 1. [file elife-92709-fig1-data1.zip › Figure 1 - Source Data/Figure 1 - source data 2 (Panel 1C)/22.05.01_16.44.52 Annotated.TIF]

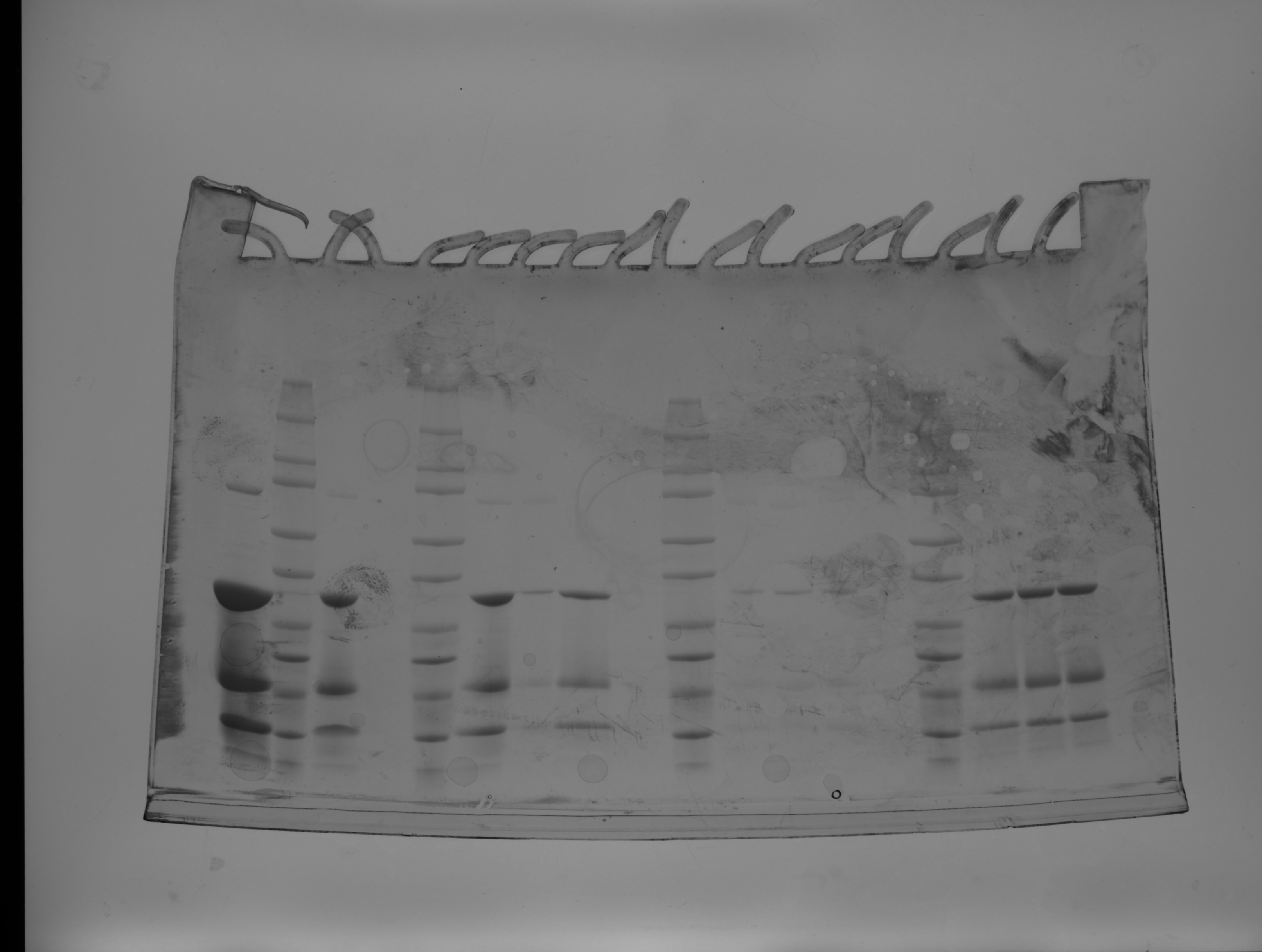

Supplement: Figure 1—source data 1. [file elife-92709-fig1-data1.zip › Figure 1 - Source Data/Figure 1 - source data 2 (Panel 1C)/22.05.01_16.44.52.tif]

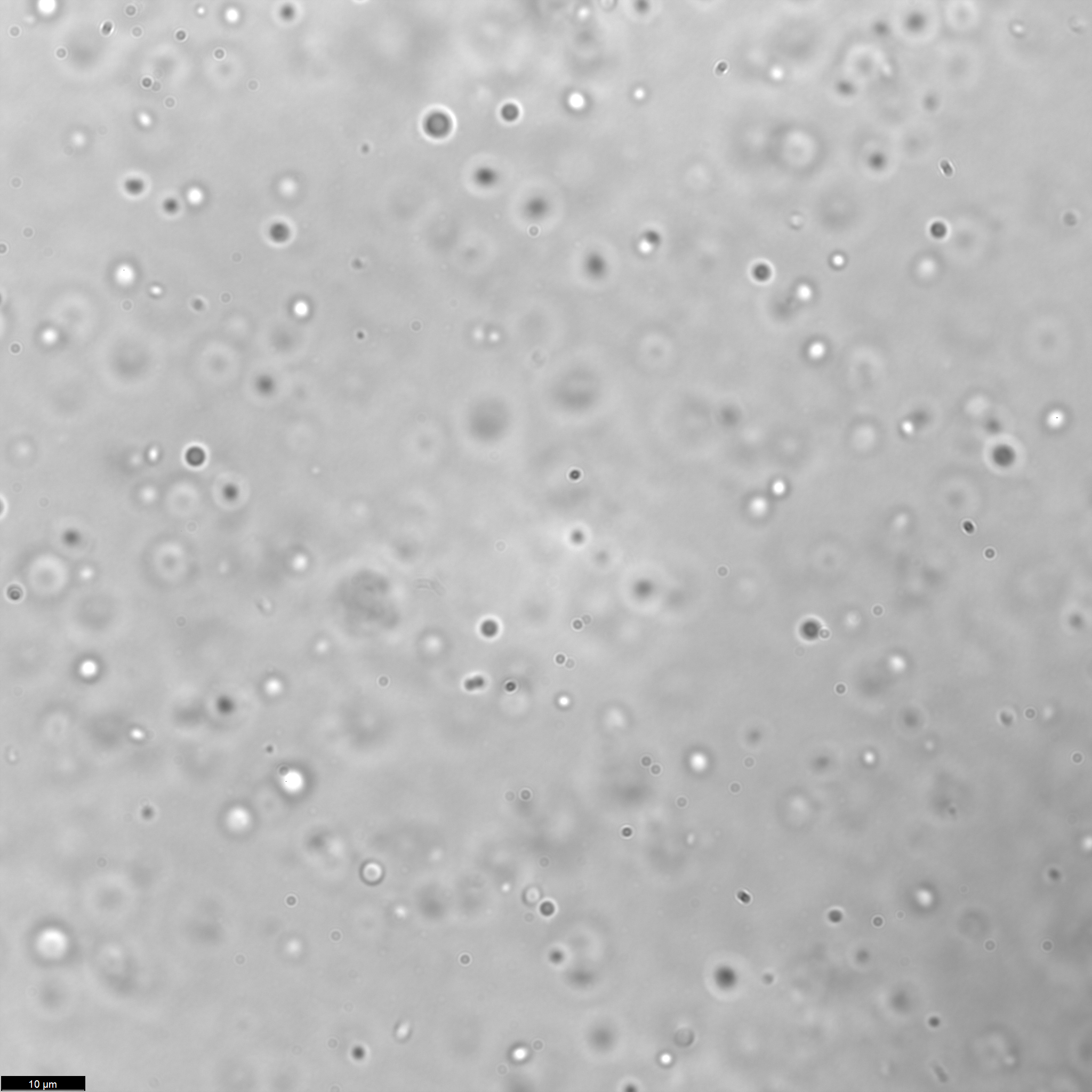

Supplement: Figure 1—source data 1. [file elife-92709-fig1-data1.zip › Figure 1 - Source Data/Figure 1 - source data 3 (Panel 1E)/20210826_WTISB_DropletAssay_Image025_ch00 - 10 second.tif]

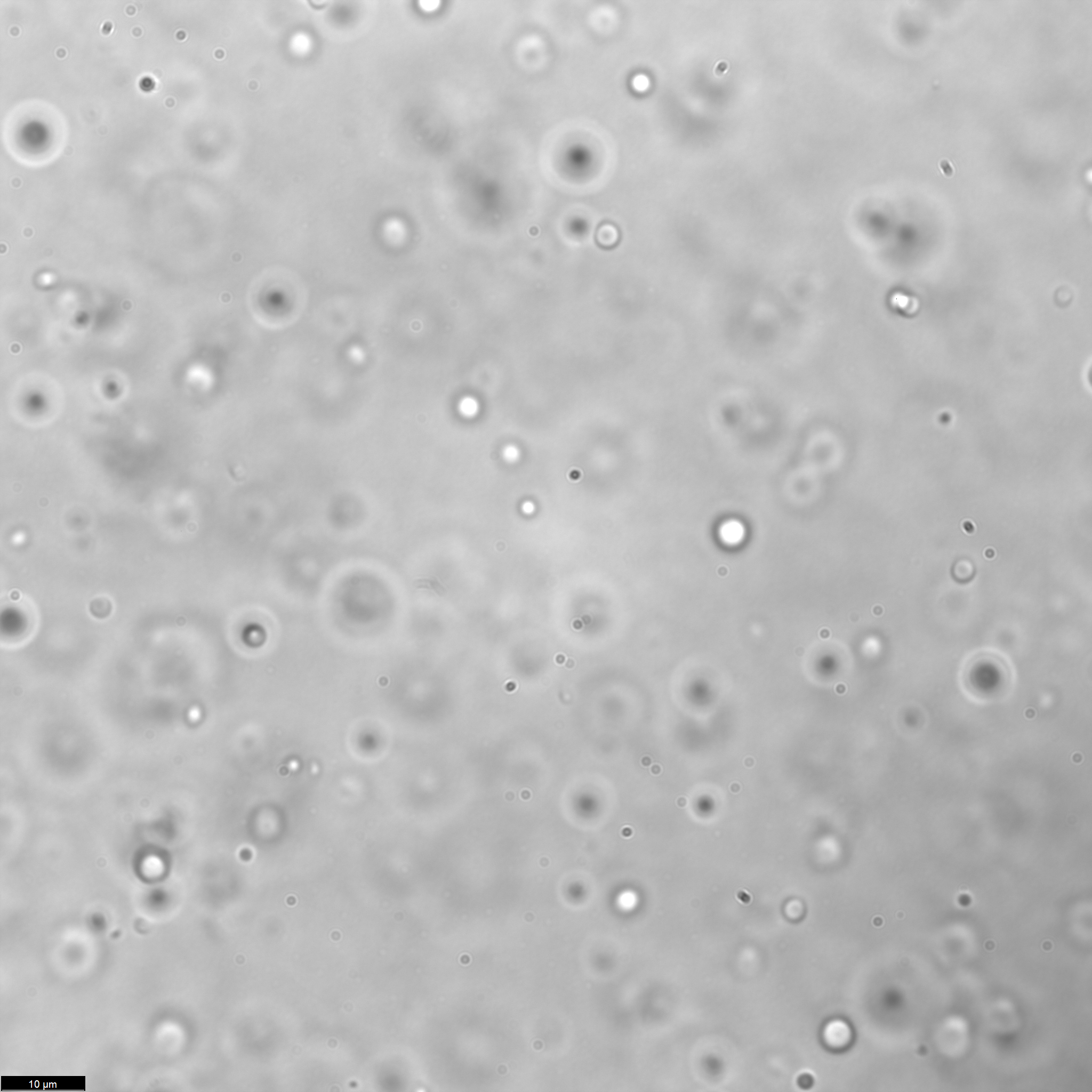

Supplement: Figure 1—source data 1. [file elife-92709-fig1-data1.zip › Figure 1 - Source Data/Figure 1 - source data 3 (Panel 1E)/20210826_WTISB_DropletAssay_Image026_ch00 - 100 seconds.tif]

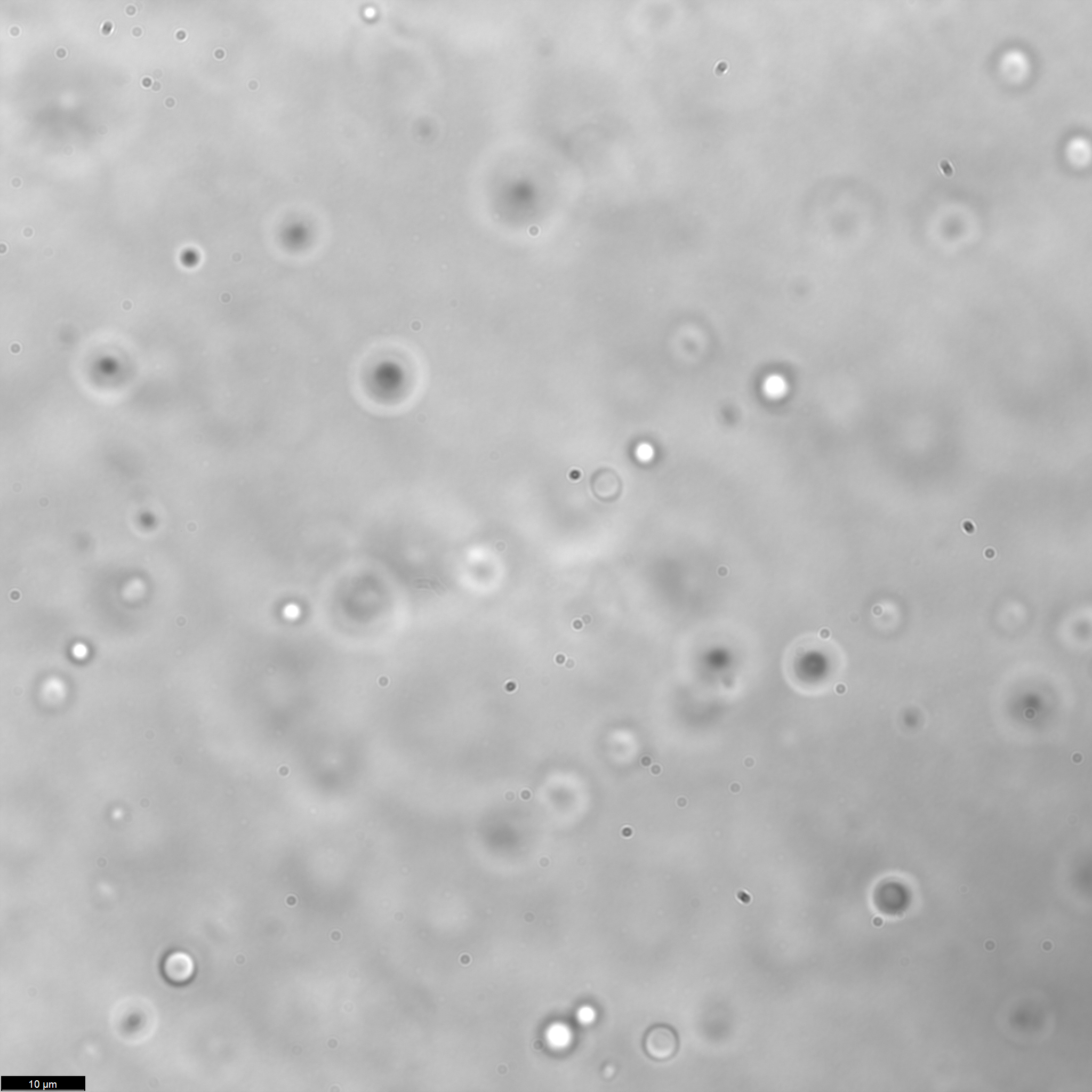

Supplement: Figure 1—source data 1. [file elife-92709-fig1-data1.zip › Figure 1 - Source Data/Figure 1 - source data 3 (Panel 1E)/20210826_WTISB_DropletAssay_Image027_ch00 - 300 seconds.tif]

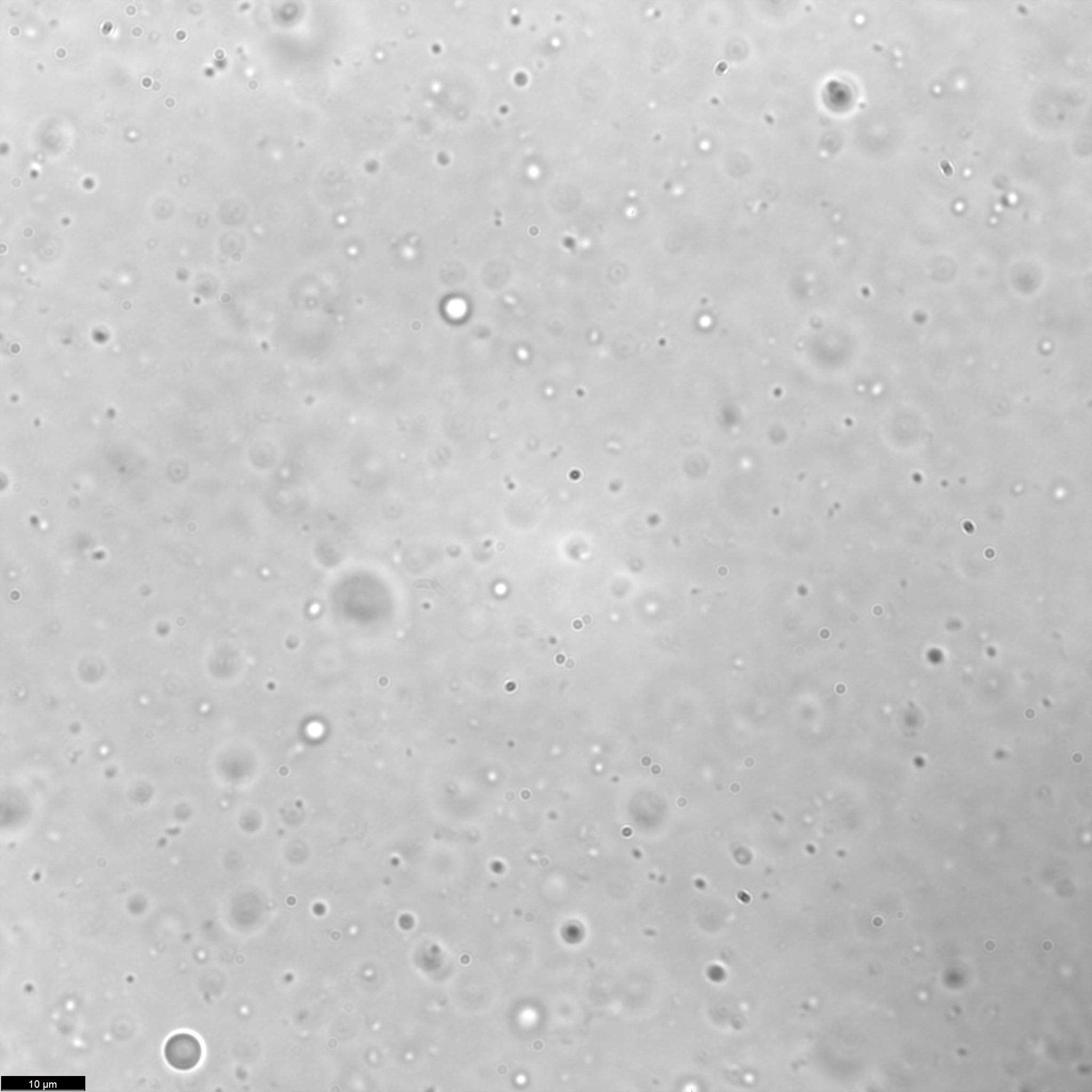

Supplement: Figure 1—source data 1. [file elife-92709-fig1-data1.zip › Figure 1 - Source Data/Figure 1 - source data 3 (Panel 1E)/20210826_WTISB_DropletAssay_Image028_ch00 - 1000 seconds.tif]

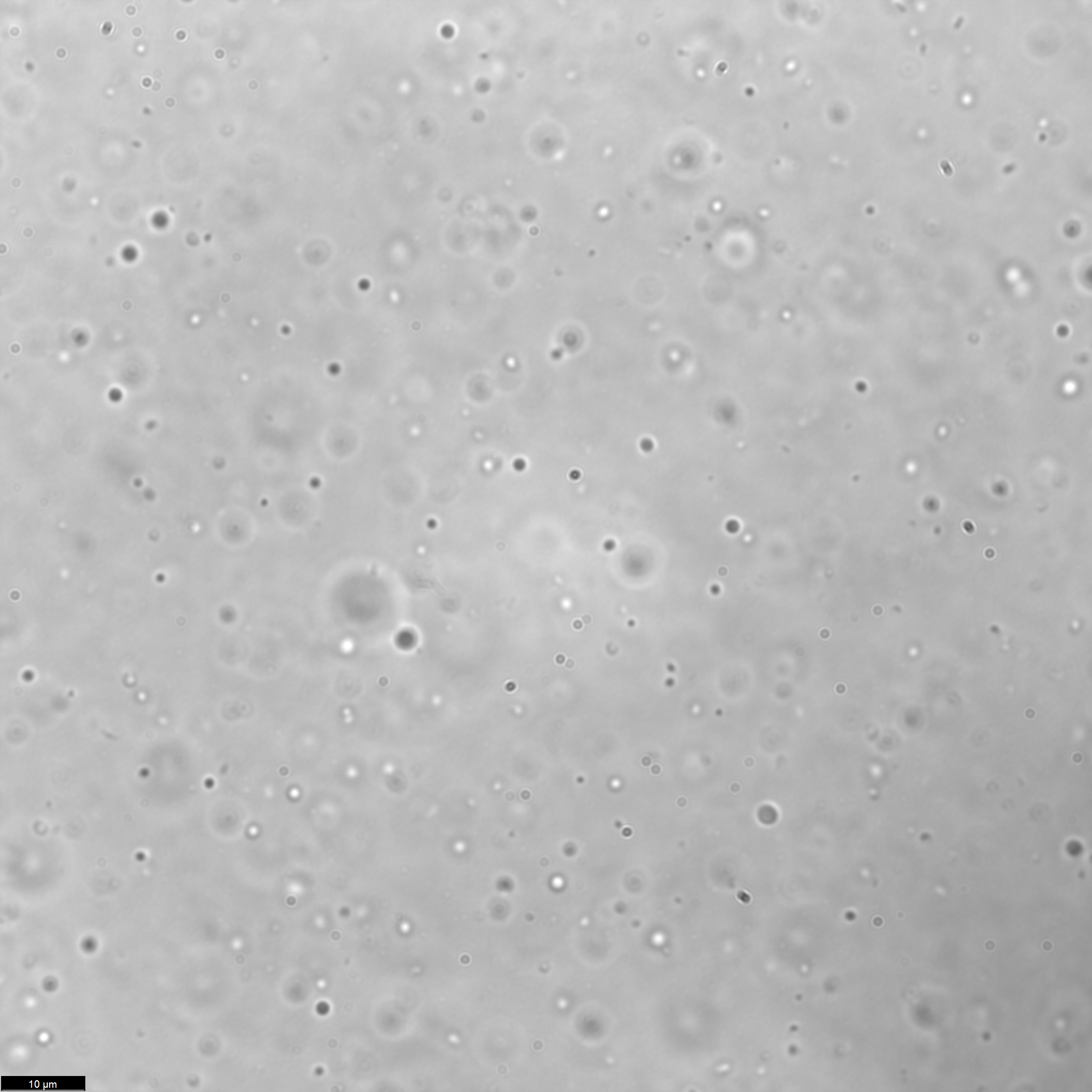

Supplement: Figure 1—source data 1. [file elife-92709-fig1-data1.zip › Figure 1 - Source Data/Figure 1 - source data 3 (Panel 1E)/20210826_WTISB_DropletAssay_Image030_ch00 - 3000 seconds.tif]

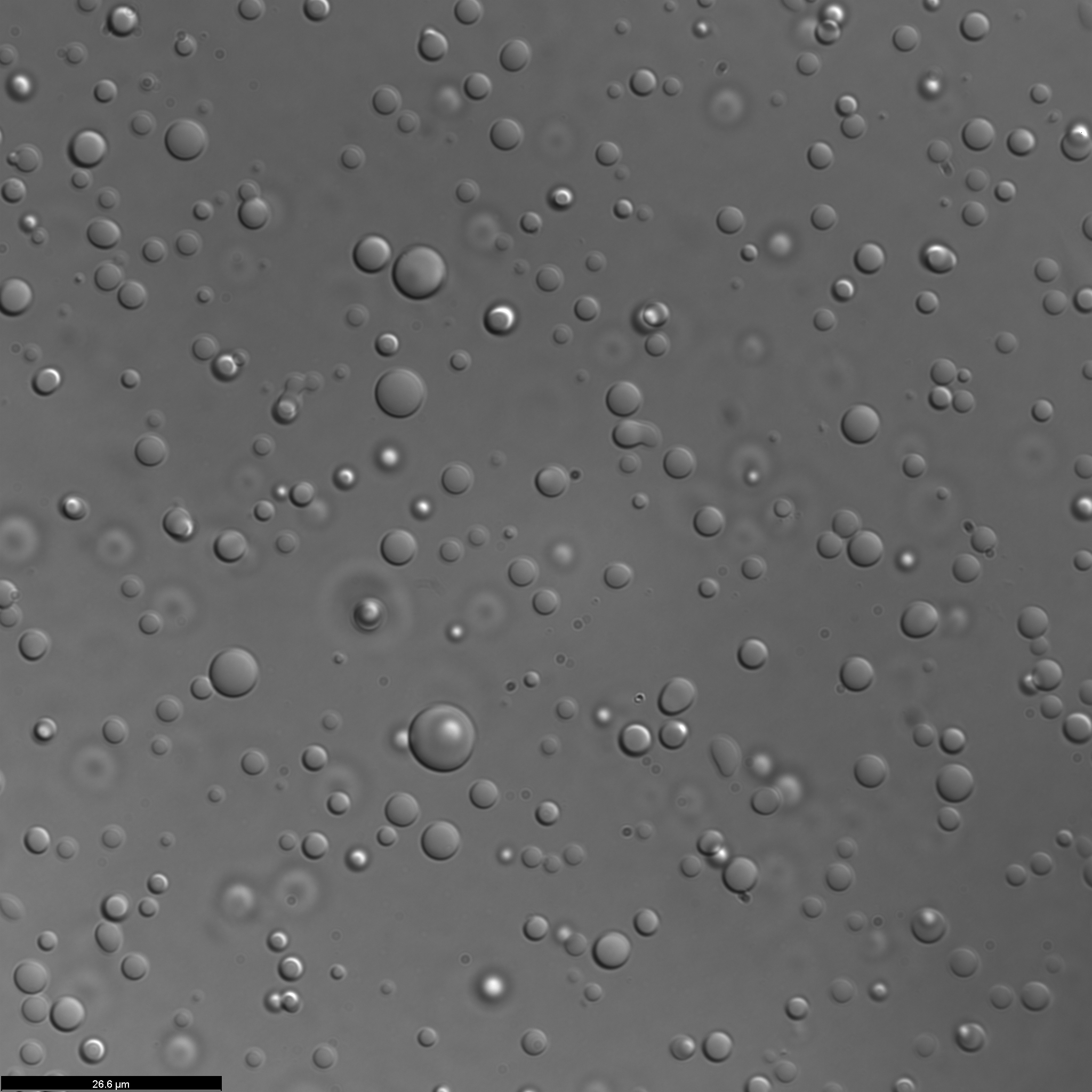

Supplement: Figure 3—source data 1. [file elife-92709-fig3-data1.zip › Figure 3 - Source Data/Figure 3 - source data 1 (Panel C)/20210507_ISB_TDP43_DropletAssay_WT Space 3 bottom_ch00.tif]

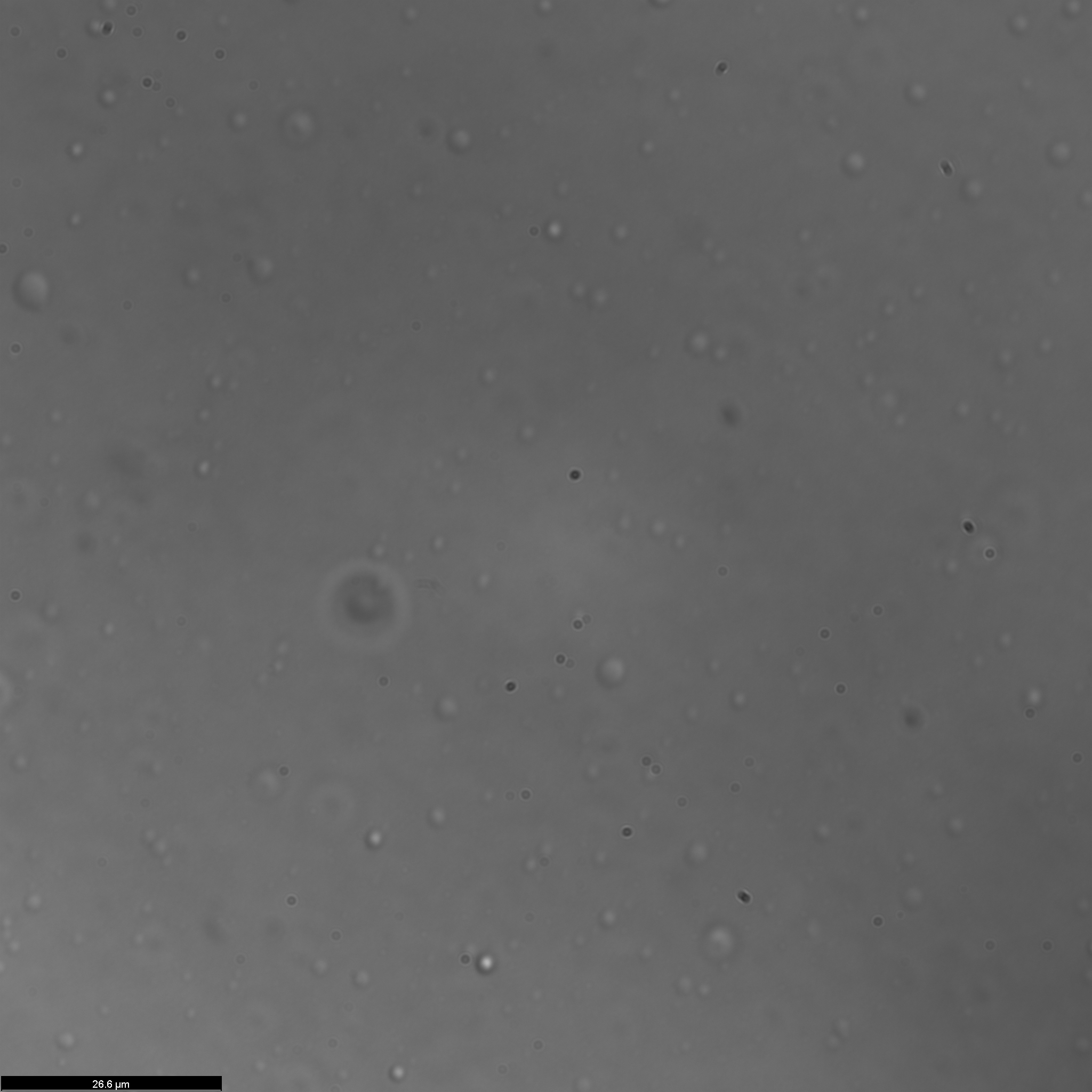

Supplement: Figure 3—source data 1. [file elife-92709-fig3-data1.zip › Figure 3 - Source Data/Figure 3 - source data 1 (Panel C)/20210507_ISB_TDP43_DropletAssay_5R space 2 bottom_ch00.tif]

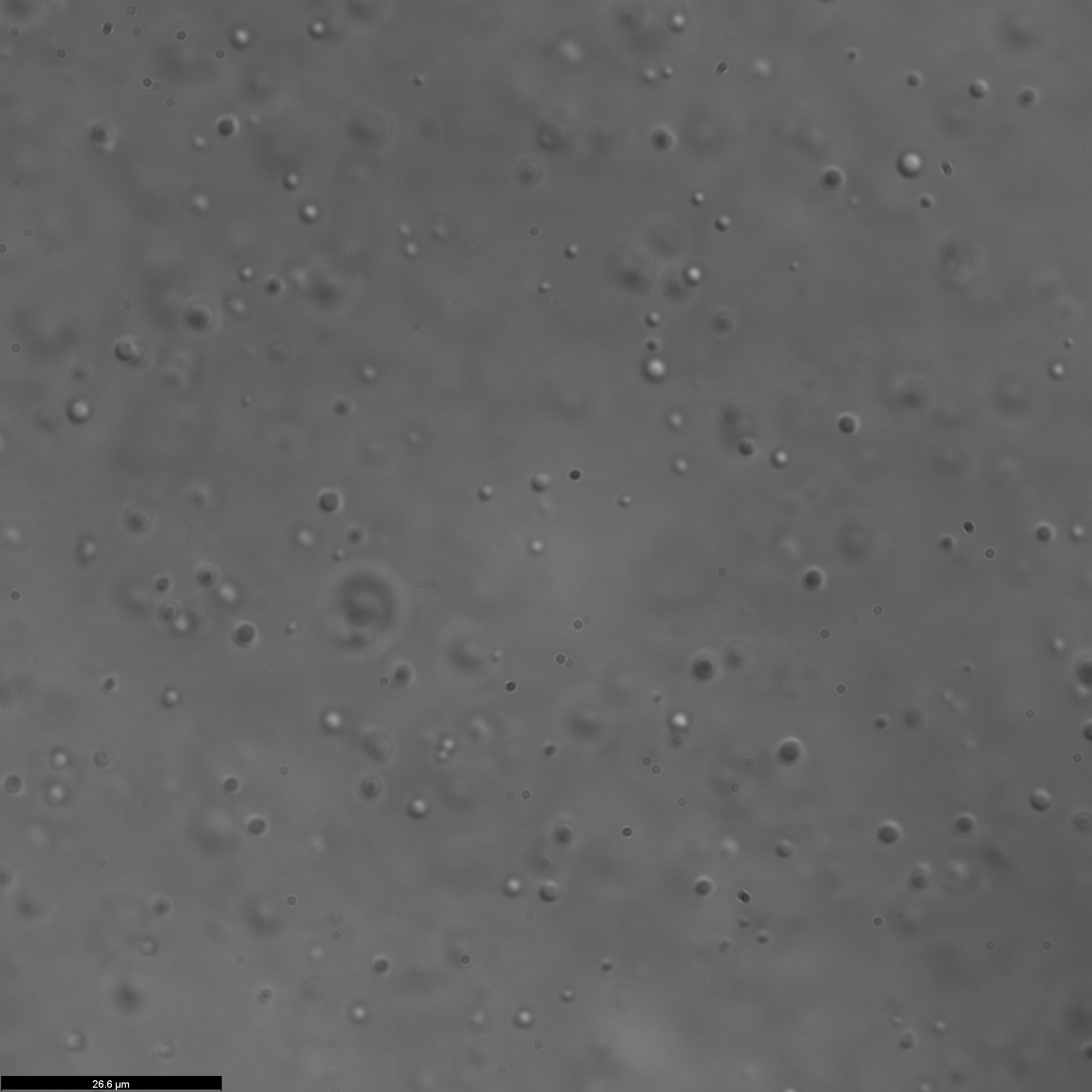

Supplement: Figure 3—source data 1. [file elife-92709-fig3-data1.zip › Figure 3 - Source Data/Figure 3 - source data 1 (Panel C)/20210507_ISB_TDP43_DropletAssay_5A Space 4 bottom_ch00.tif]

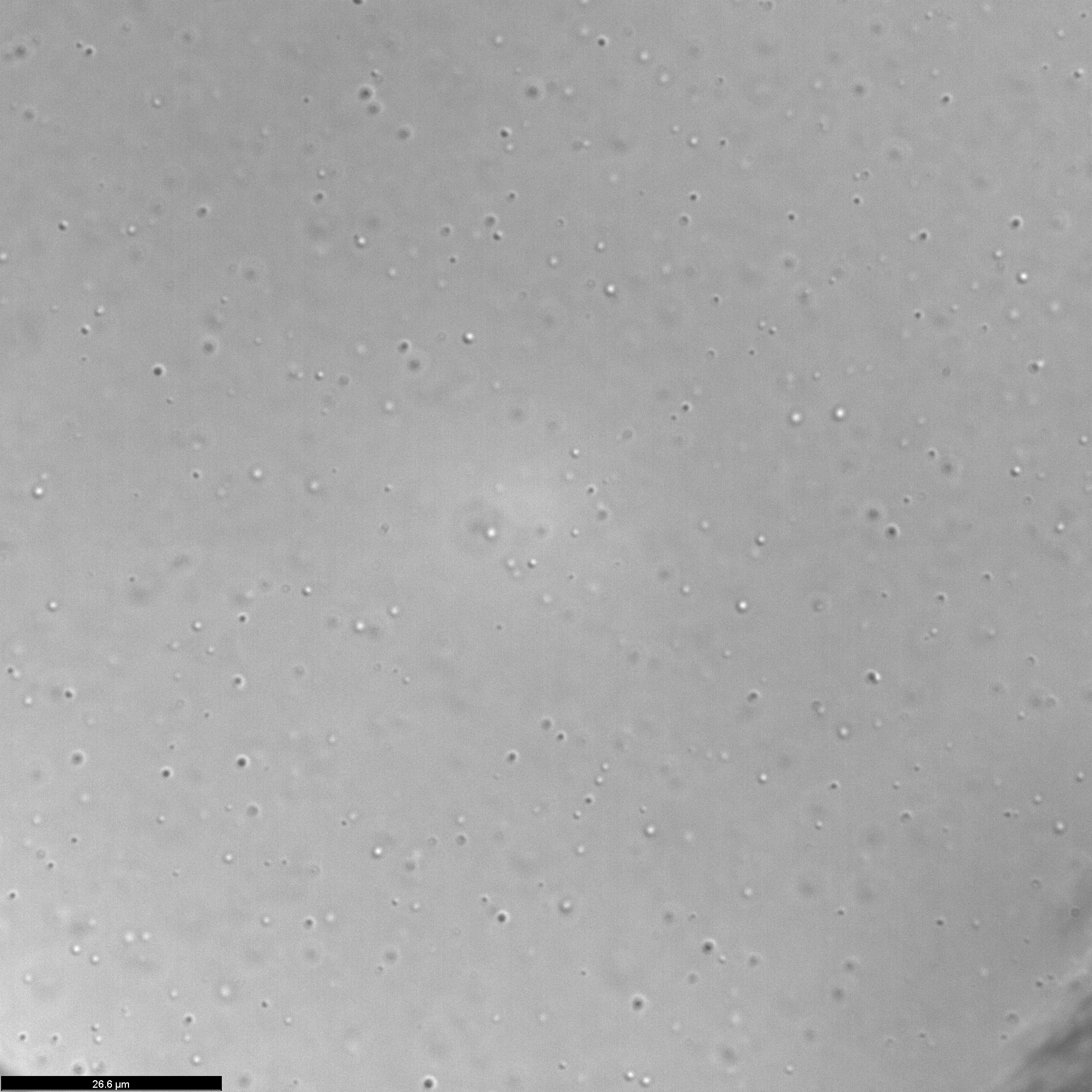

Supplement: Figure 4—source data 1. [file elife-92709-fig4-data1.zip › Figure 4 - Source Data/Figure 4 - source data 1 (Panel C)/20211208 Turbidity Assay_ISB 4R 75 mM NaCl 1_ch00.tif]

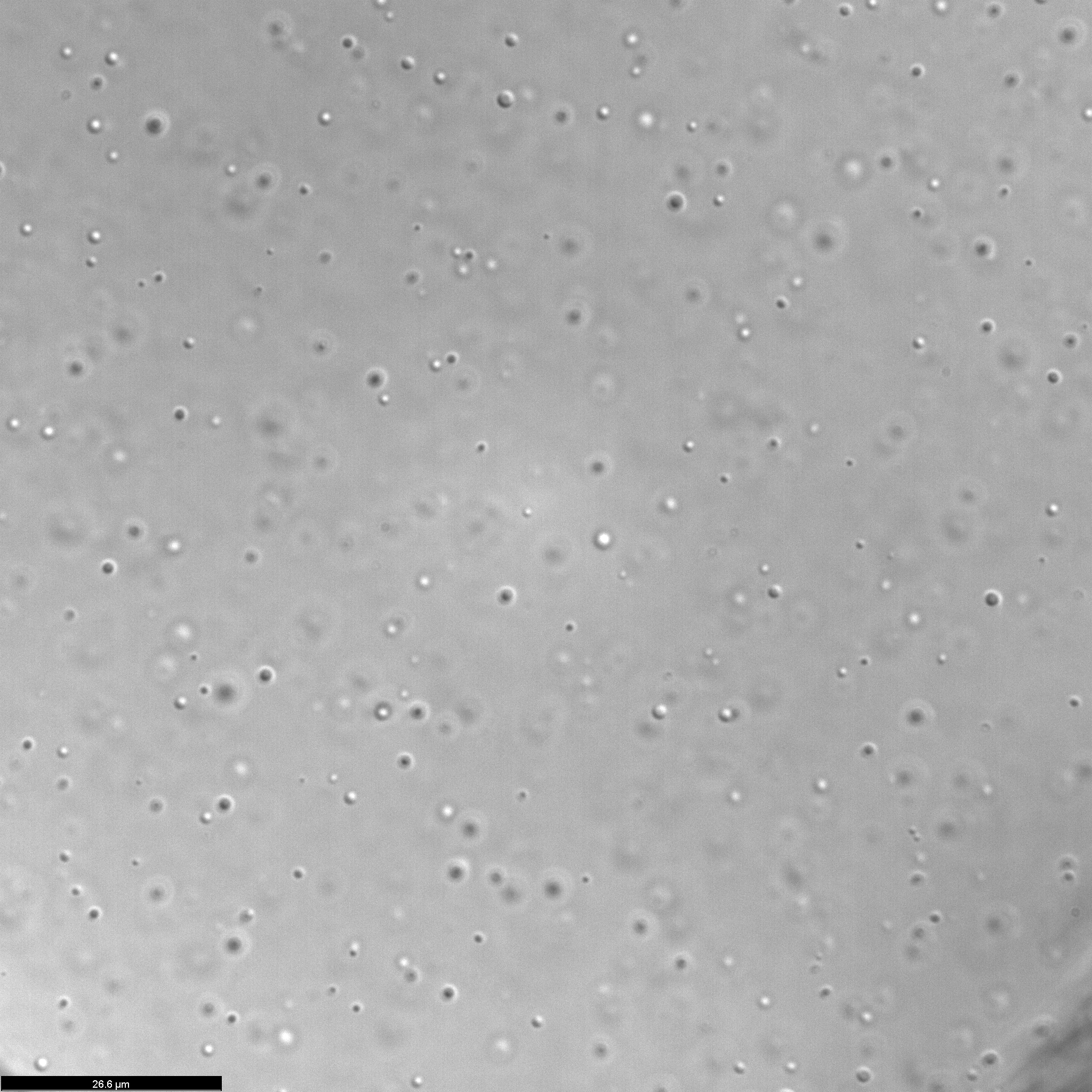

Supplement: Figure 4—source data 1. [file elife-92709-fig4-data1.zip › Figure 4 - Source Data/Figure 4 - source data 1 (Panel C)/20211208 Turbidity Assay_ISB E2 75 mM NaCl 2_ch00.tif]

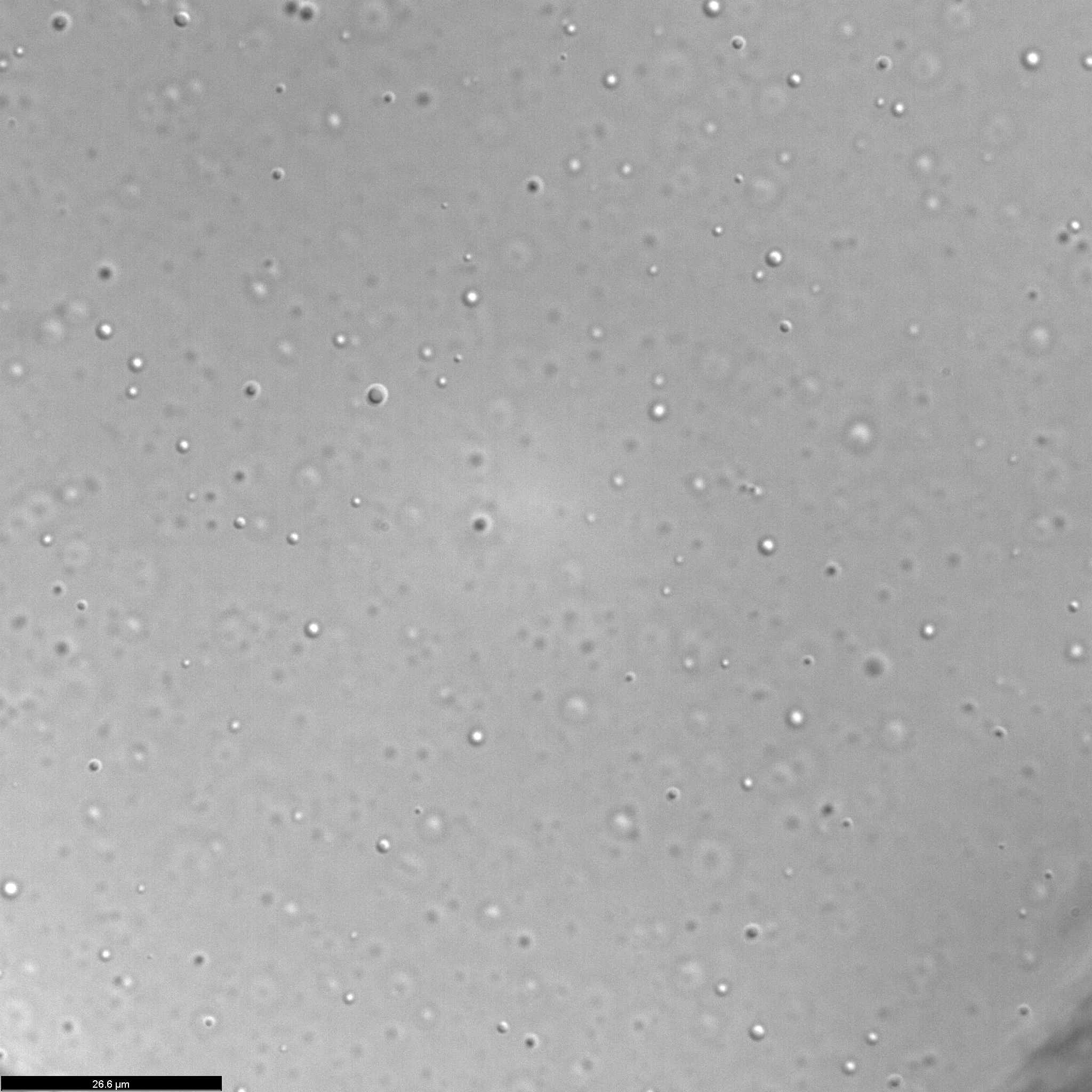

Supplement: Figure 4—source data 1. [file elife-92709-fig4-data1.zip › Figure 4 - Source Data/Figure 4 - source data 1 (Panel C)/20211208 Turbidity Assay_ISB E3 75 mM NaCl 2_ch00.tif]

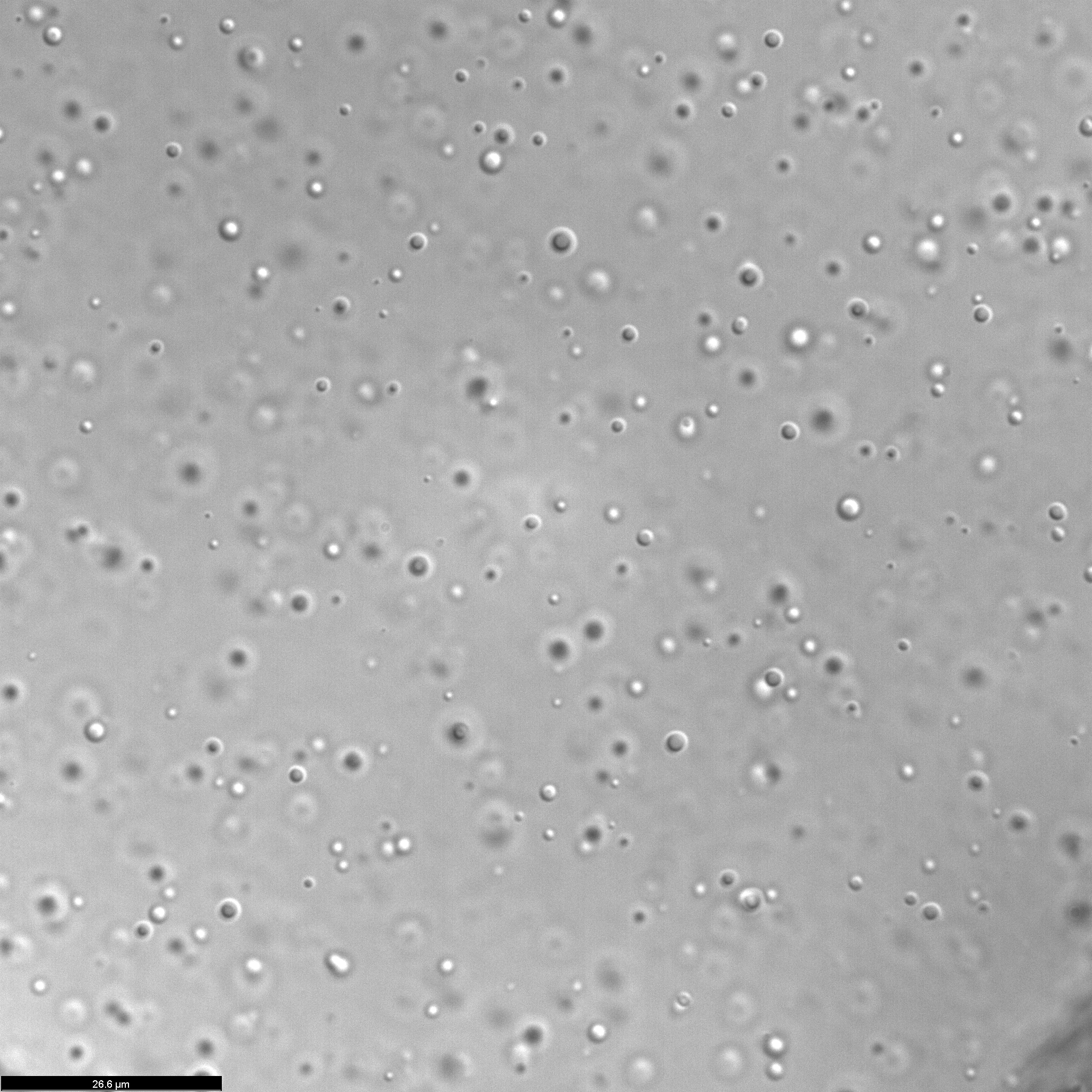

Supplement: Figure 4—source data 1. [file elife-92709-fig4-data1.zip › Figure 4 - Source Data/Figure 4 - source data 1 (Panel C)/20211208 Turbidity Assay_WT ISB 75 mM NaCl 1_ch00.tif]

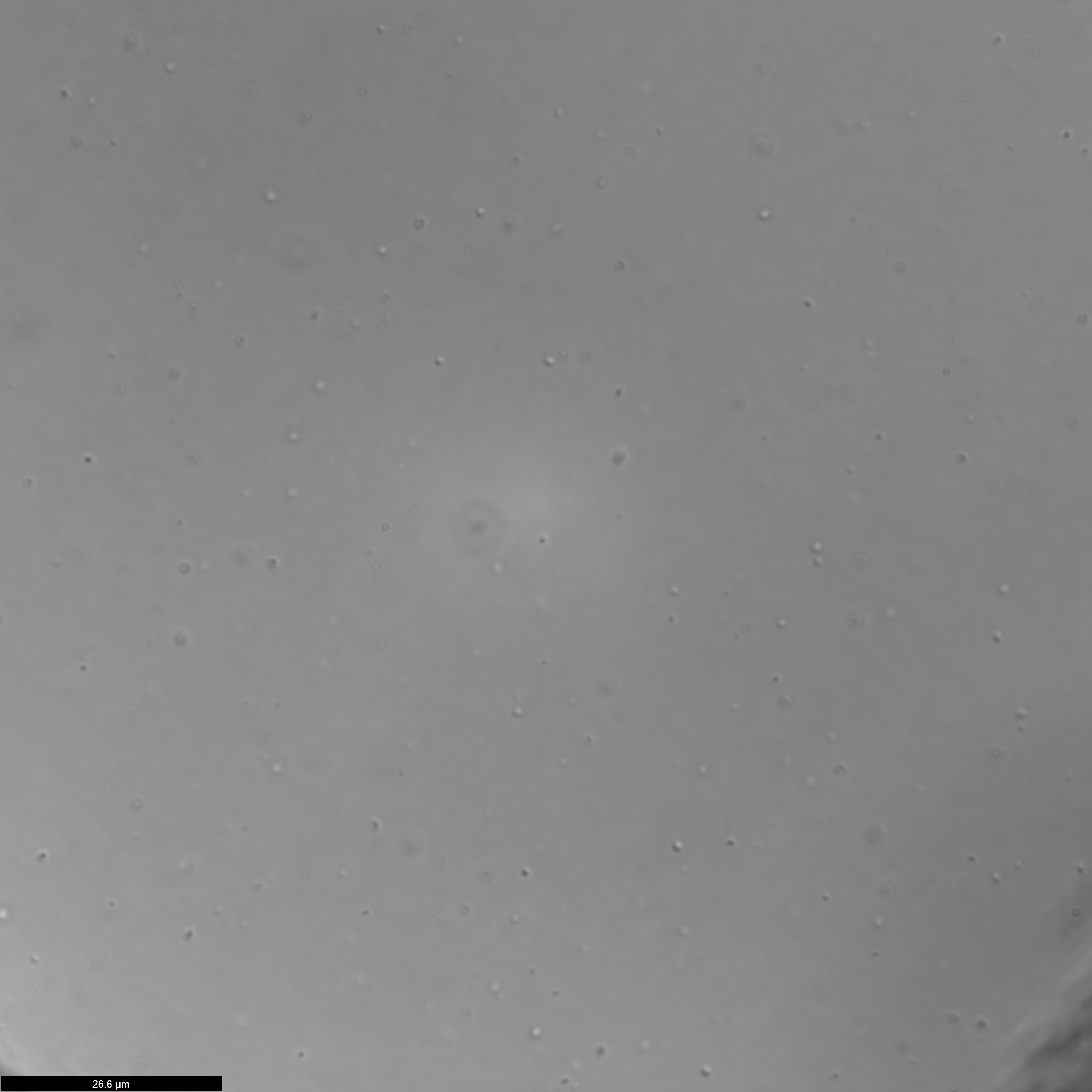

Supplement: Figure 4—source data 1. [file elife-92709-fig4-data1.zip › Figure 4 - Source Data/Figure 4 - source data 3 (Panel F)/ISB Reversal Mutants Droplet Test 20220303_ISB R40E 2_ch00.tif]

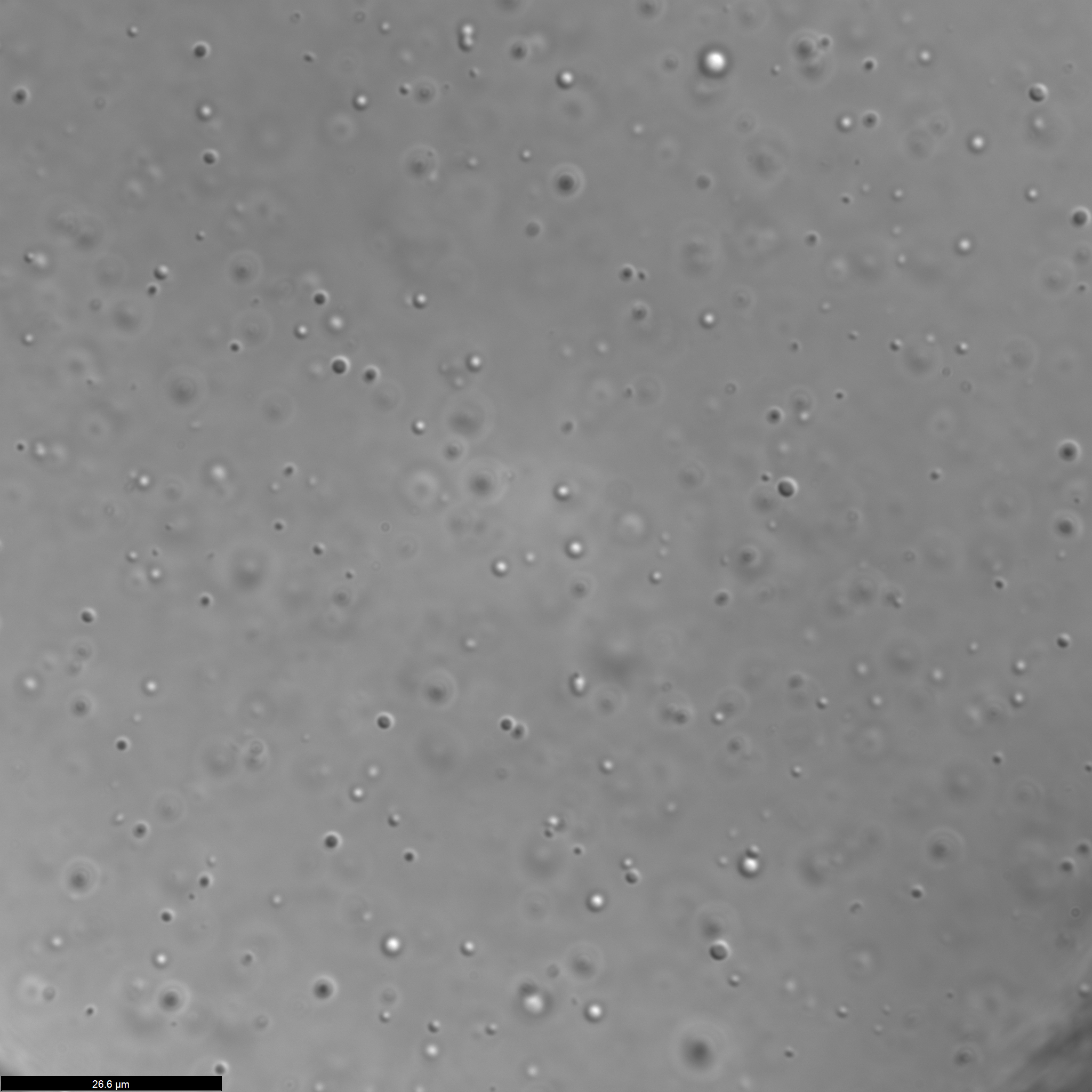

Supplement: Figure 4—source data 1. [file elife-92709-fig4-data1.zip › Figure 4 - Source Data/Figure 4 - source data 3 (Panel F)/ISB Reversal Mutants Droplet Test 20220303_ISB R42E 2_ch00.tif]

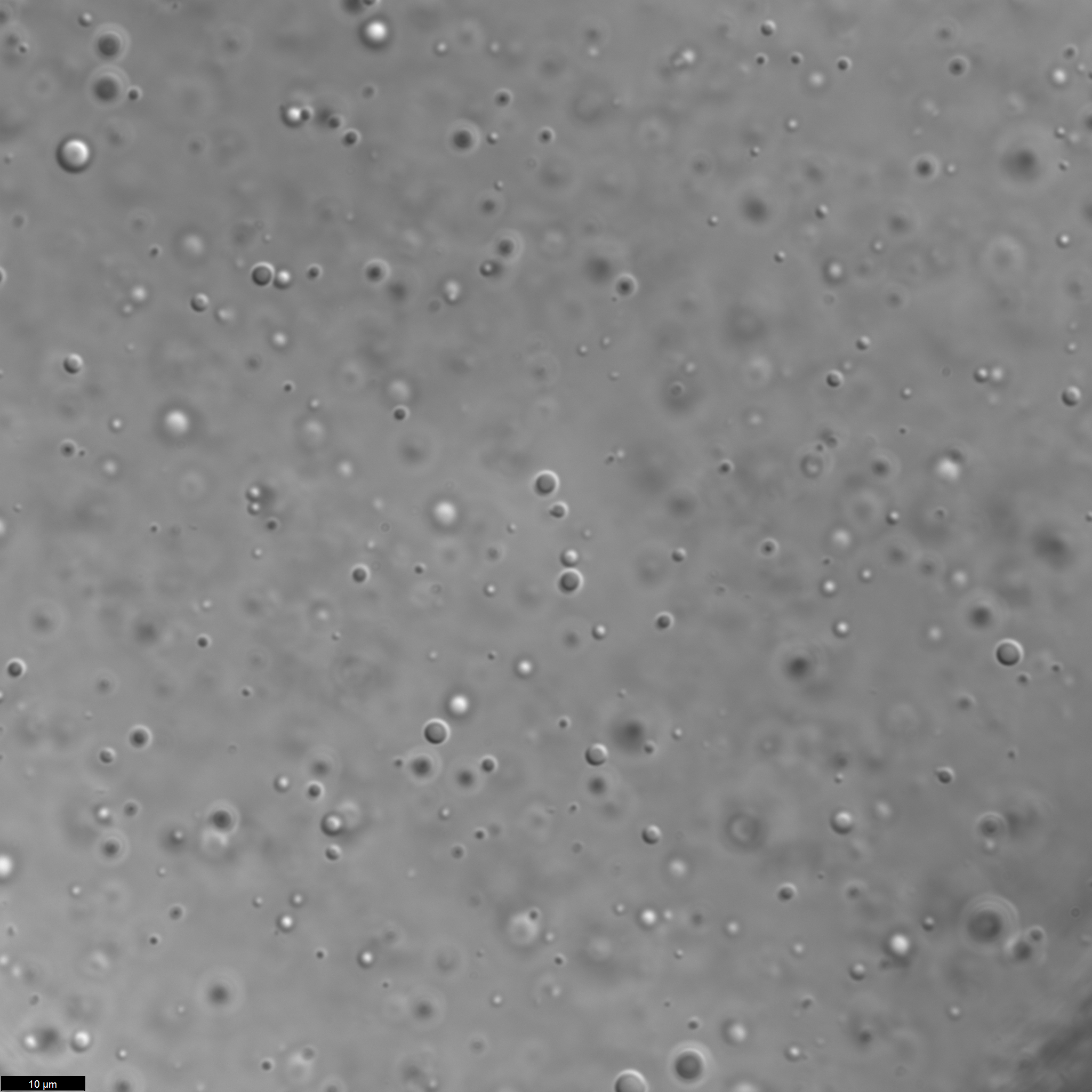

Supplement: Figure 4—source data 1. [file elife-92709-fig4-data1.zip › Figure 4 - Source Data/Figure 4 - source data 3 (Panel F)/ISB Reversal Mutants Droplet Test 20220303_ISB WT_ch00 (CORRECTED ERROR BARS).tif]

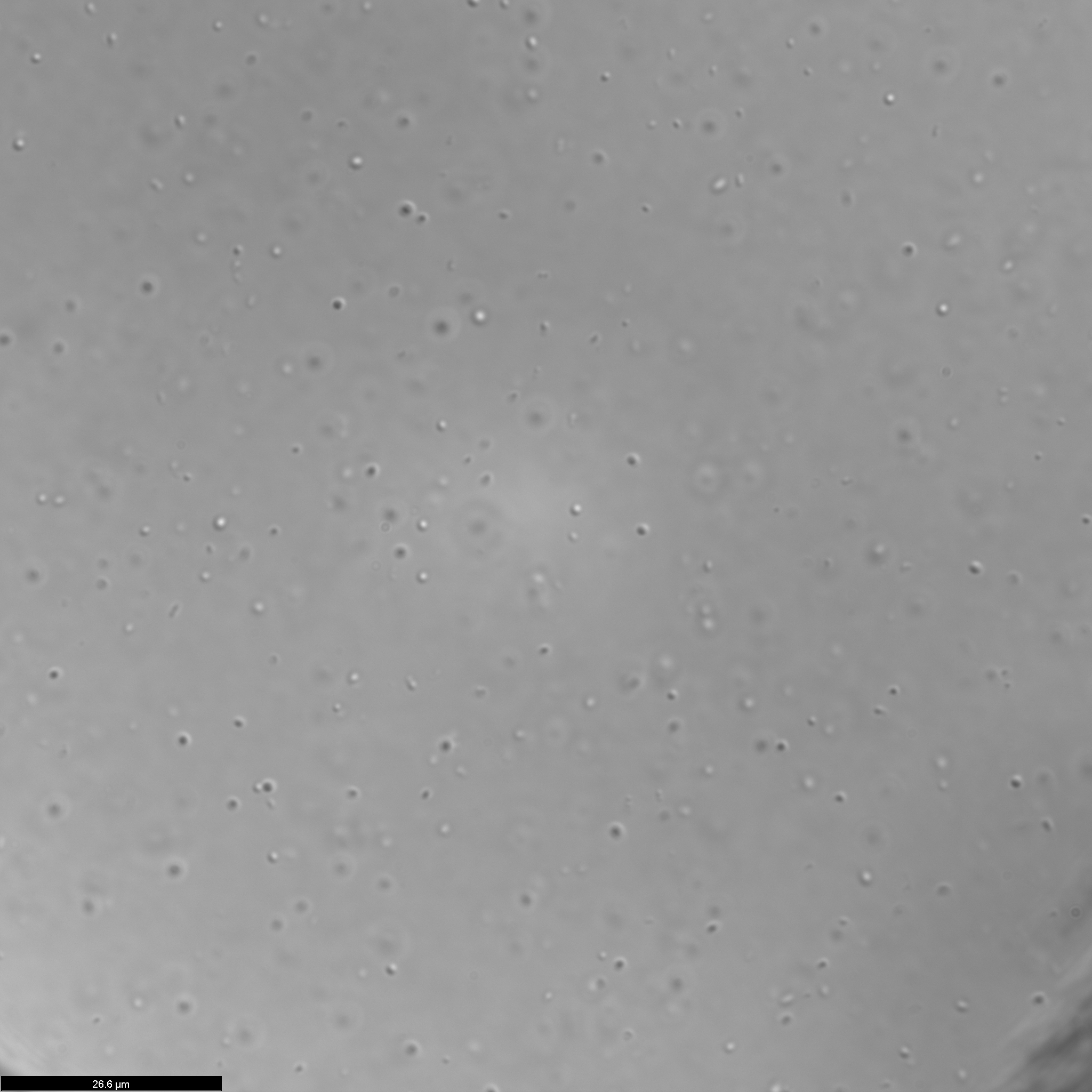

Supplement: Figure 5—source data 1. [file elife-92709-fig5-data1.zip › Figure 5 - Source Data/Figure 5 - source data 1 (Panel C)/20220412 ISB Final Mutants Droplet Test_dBorealin Image 1_ch00.tif]

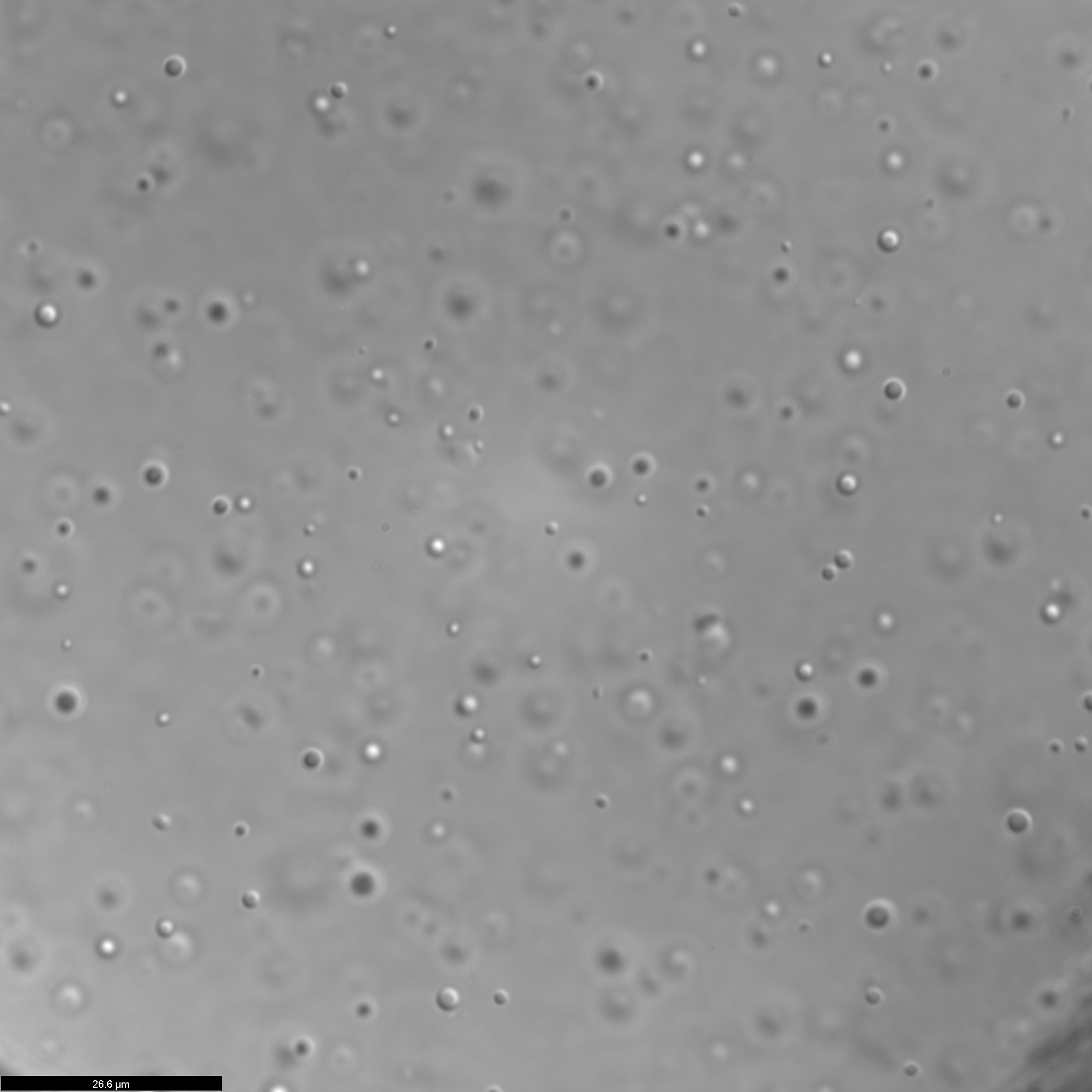

Supplement: Figure 5—source data 1. [file elife-92709-fig5-data1.zip › Figure 5 - Source Data/Figure 5 - source data 1 (Panel C)/20220412 ISB Final Mutants Droplet Test_dINCENPdBorealin Image 3_ch00.tif]

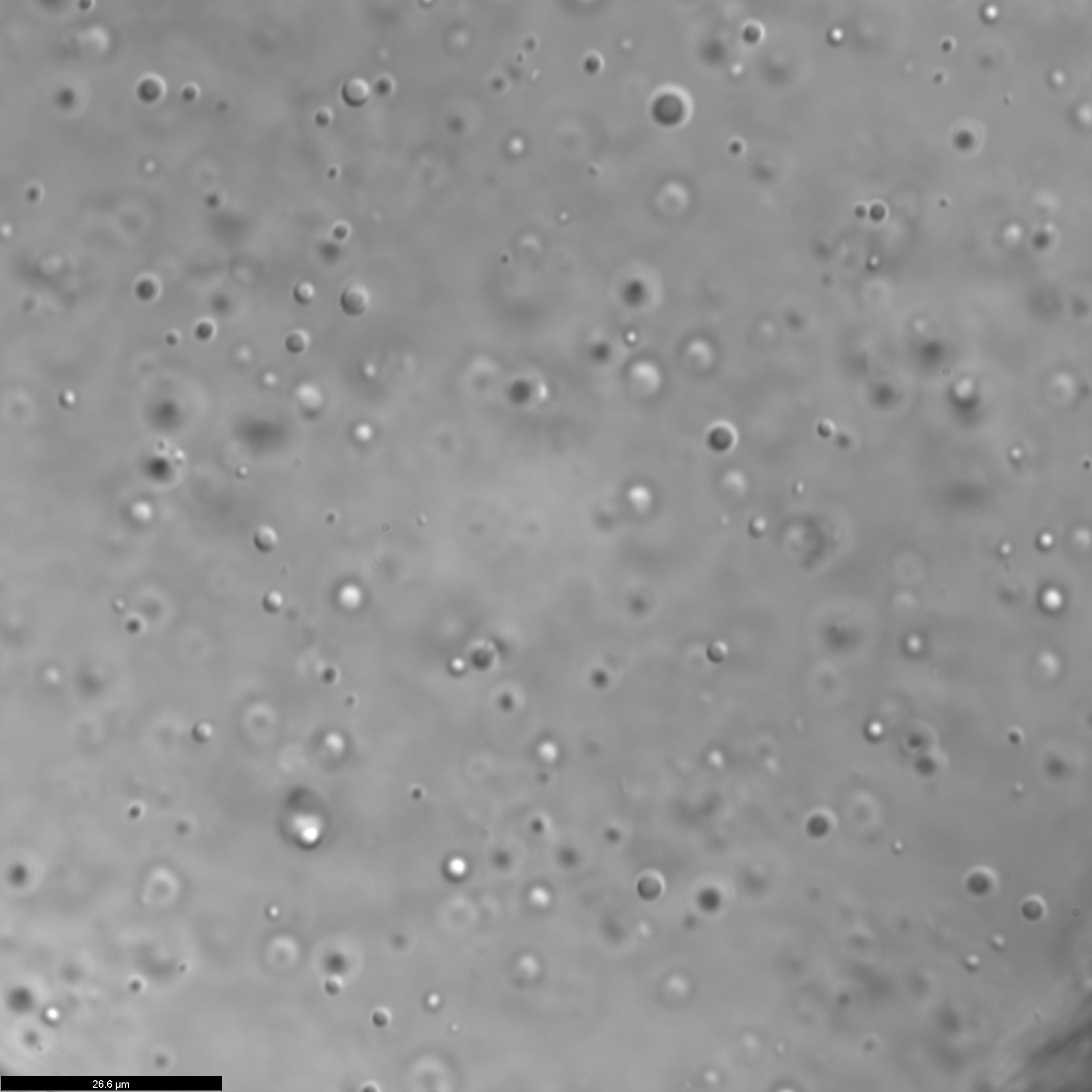

Supplement: Figure 5—source data 1. [file elife-92709-fig5-data1.zip › Figure 5 - Source Data/Figure 5 - source data 1 (Panel C)/20220412 ISB Final Mutants Droplet Test_WT Protein Image 4_ch00.tif]

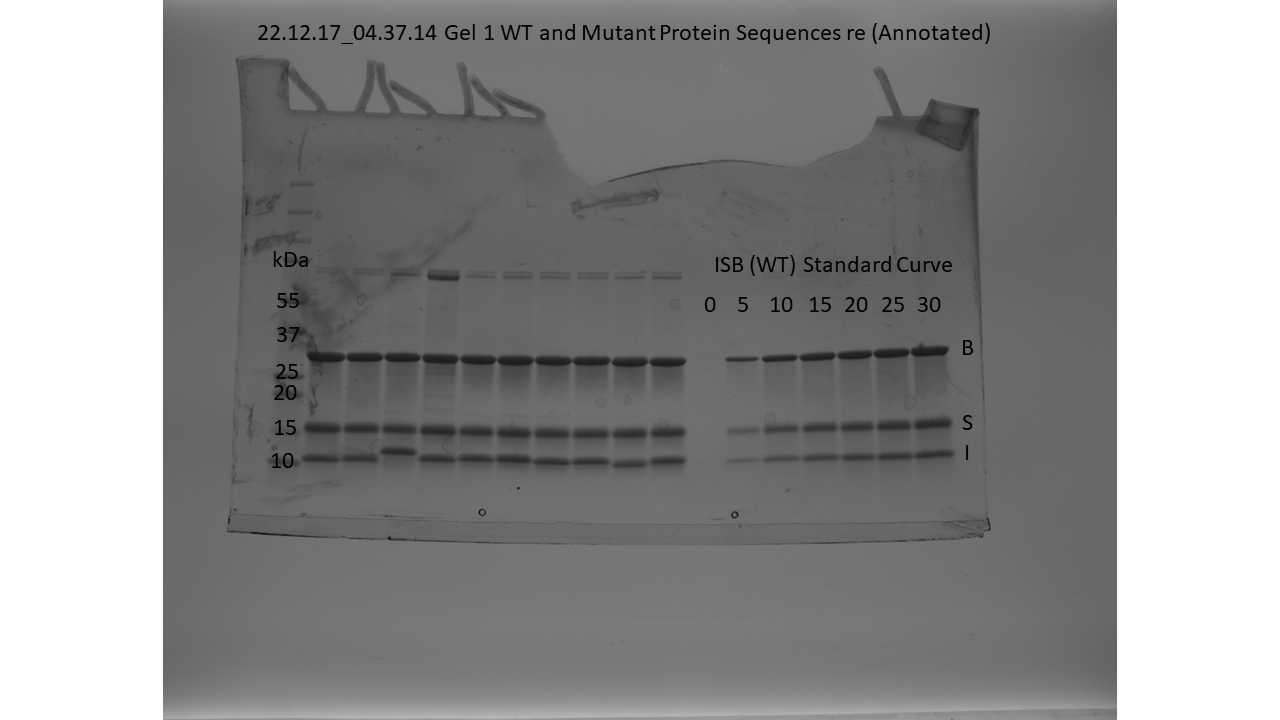

Supplement: Figure 5—figure supplement 1—source data 1. [file elife-92709-fig5-figsupp1-data1.zip › Figure 5 - figure supplement 1 Source Data/22.12.17_04.37.14 Gel 1 WT and Mutant Protein Sequences re Annotated.TIF]

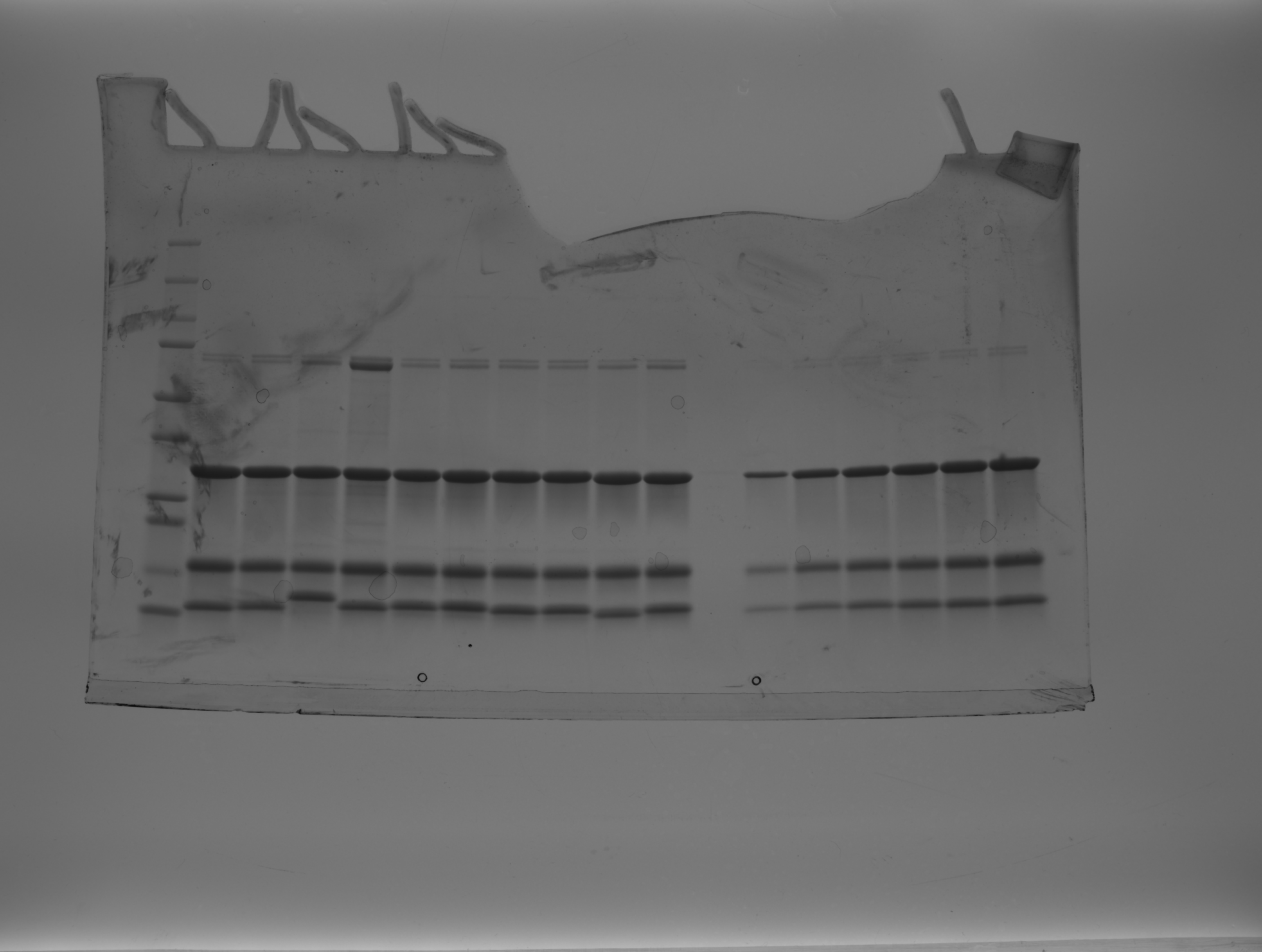

Supplement: Figure 5—figure supplement 1—source data 1. [file elife-92709-fig5-figsupp1-data1.zip › Figure 5 - figure supplement 1 Source Data/22.12.17_04.37.14 Gel 1 WT and Mutant Protein Sequences re.tif]

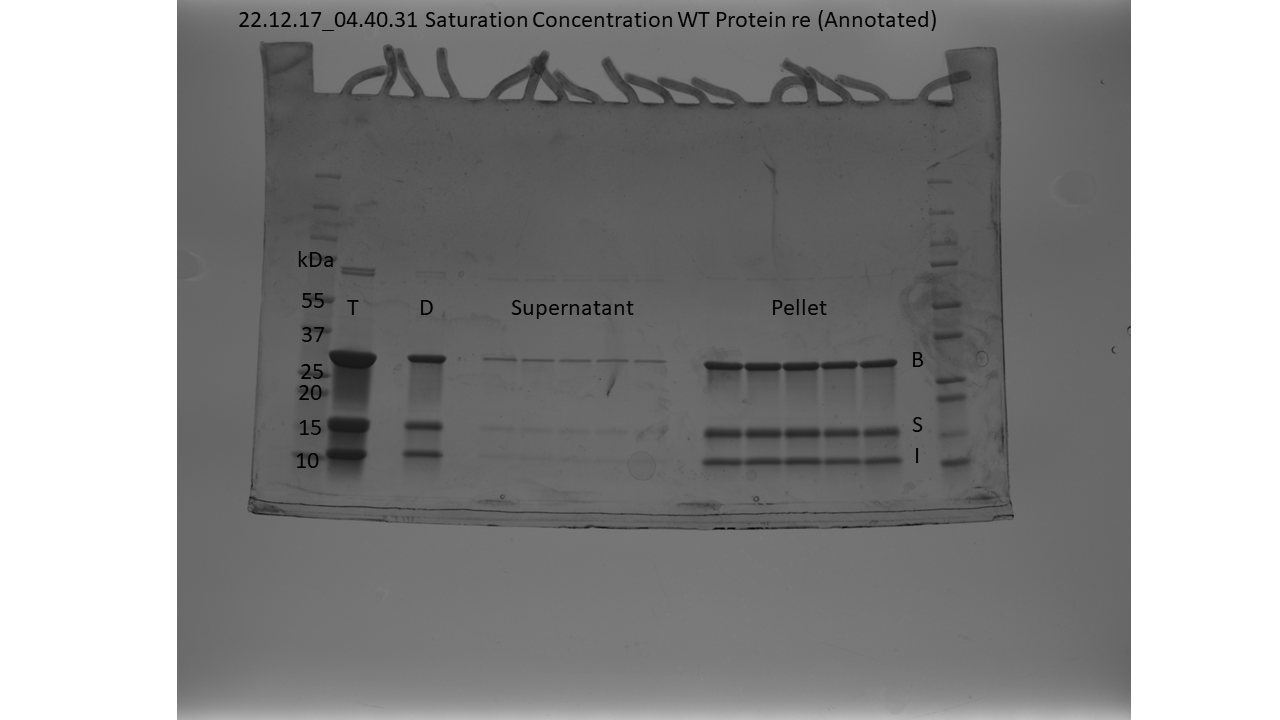

Supplement: Figure 5—figure supplement 1—source data 1. [file elife-92709-fig5-figsupp1-data1.zip › Figure 5 - figure supplement 1 Source Data/22.12.17_04.40.31 Saturation Concentration WT Protein re Annotated.TIF]

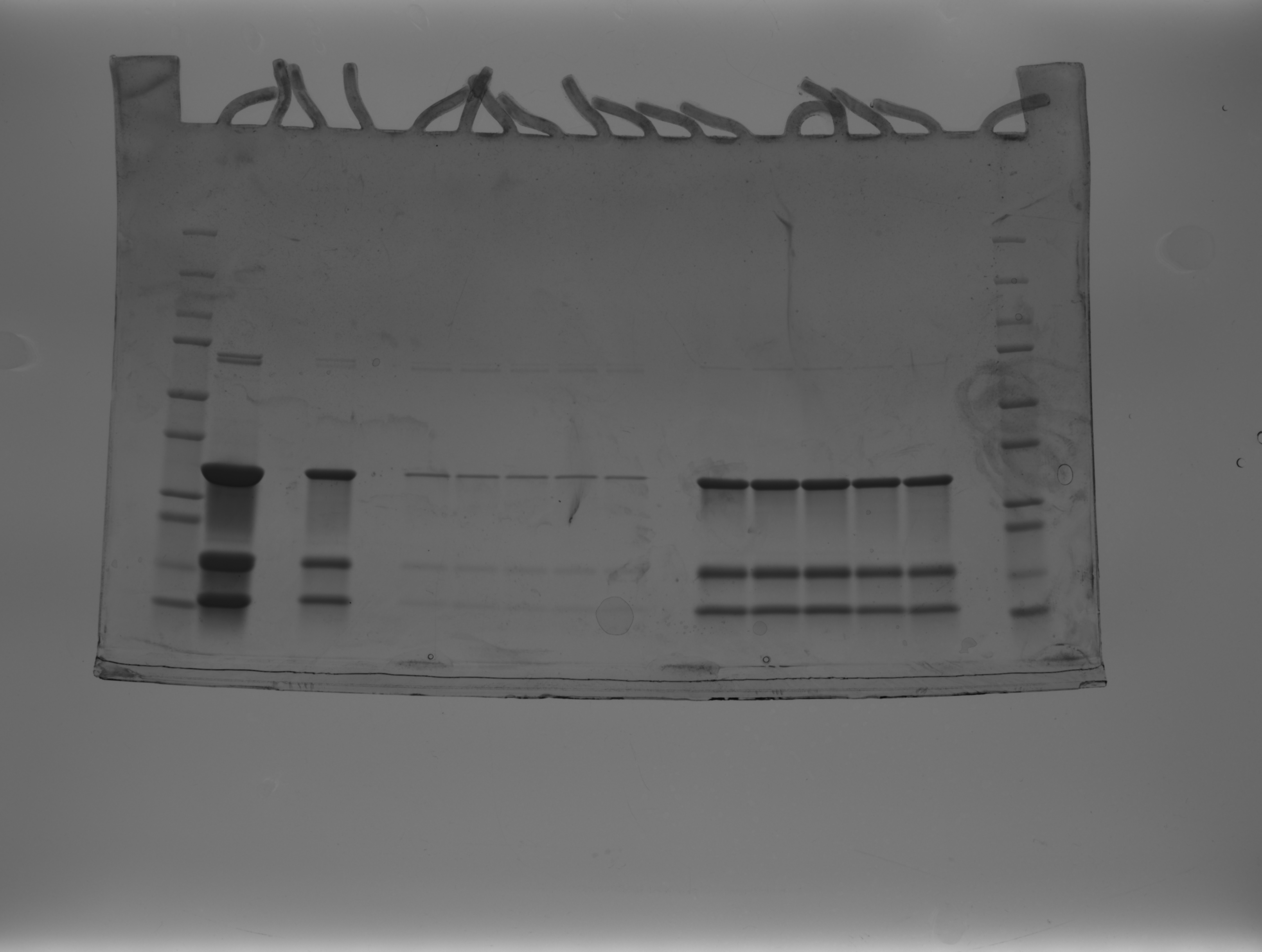

Supplement: Figure 5—figure supplement 1—source data 1. [file elife-92709-fig5-figsupp1-data1.zip › Figure 5 - figure supplement 1 Source Data/22.12.17_04.40.31 Saturation Concentration WT Protein re.tif]

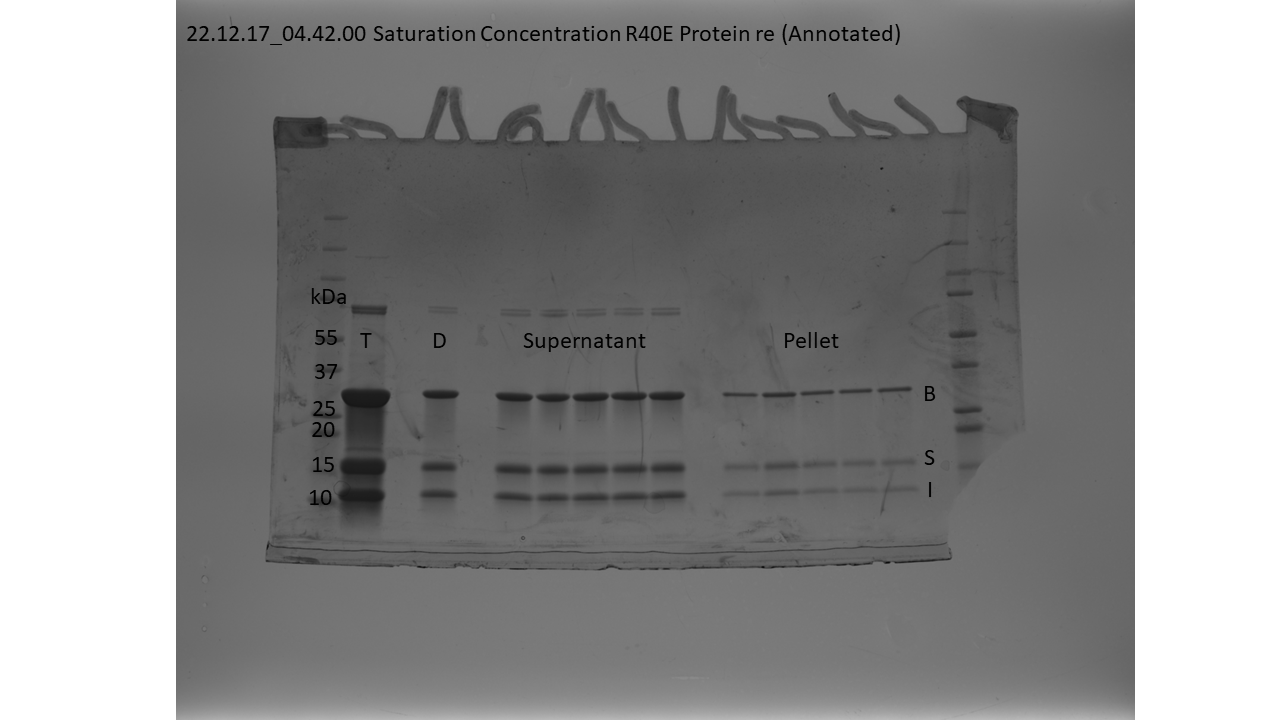

Supplement: Figure 5—figure supplement 1—source data 1. [file elife-92709-fig5-figsupp1-data1.zip › Figure 5 - figure supplement 1 Source Data/22.12.17_04.42.00 Saturation Concentration R40E Protein re Annotated.TIF]

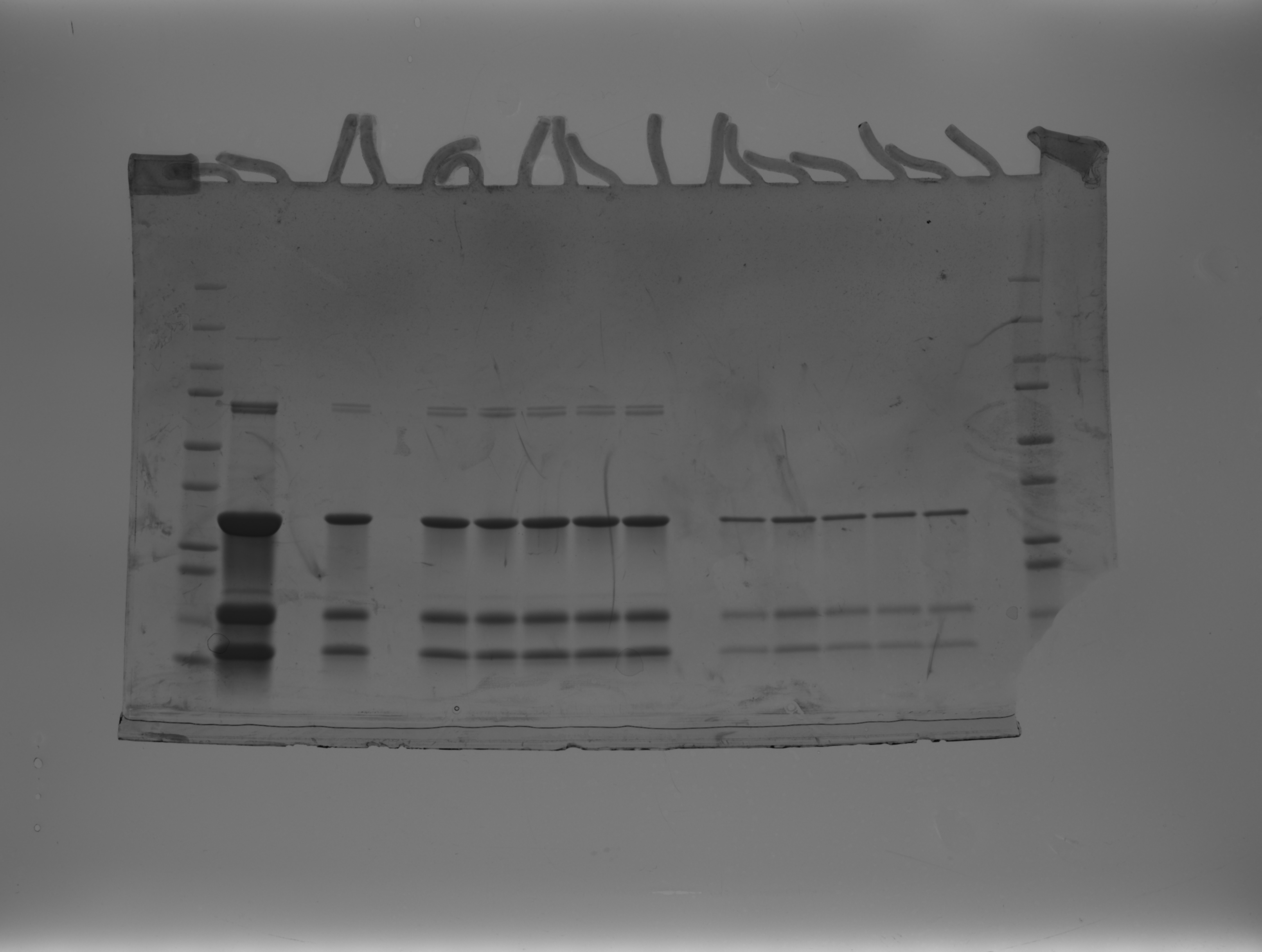

Supplement: Figure 5—figure supplement 1—source data 1. [file elife-92709-fig5-figsupp1-data1.zip › Figure 5 - figure supplement 1 Source Data/22.12.17_04.42.00 Saturation Concentration R40E Protein re.tif]

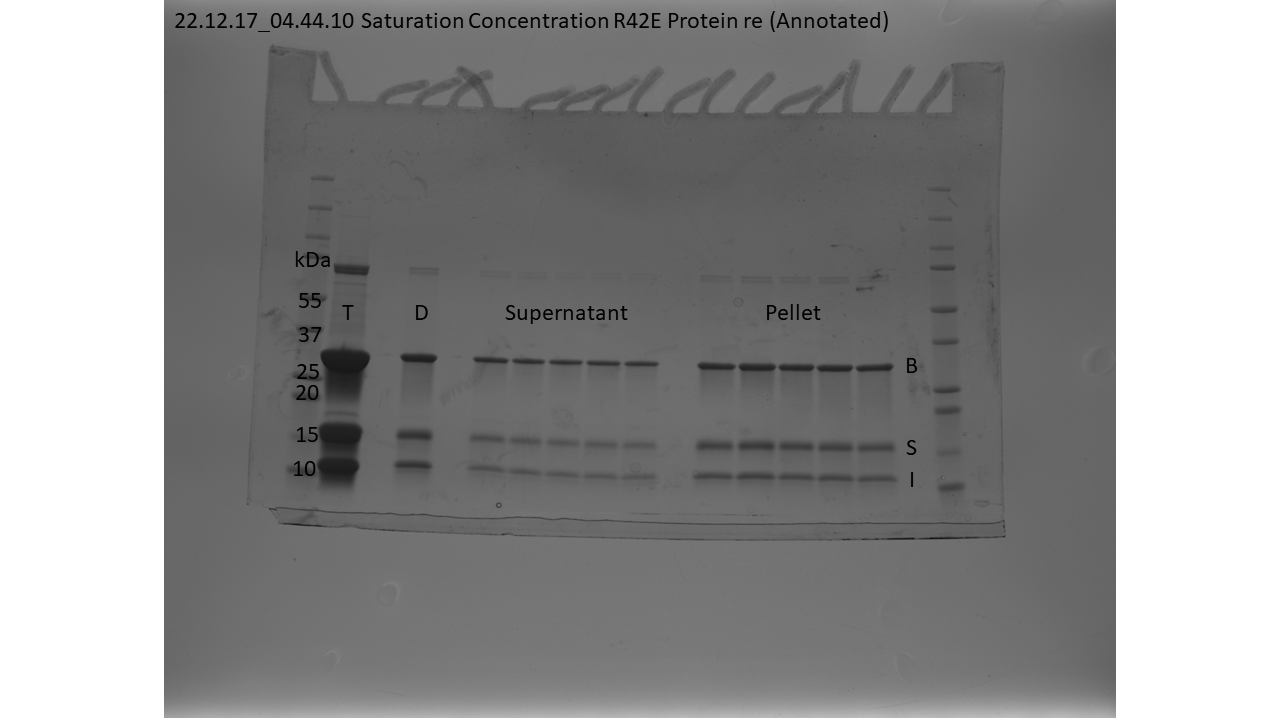

Supplement: Figure 5—figure supplement 1—source data 1. [file elife-92709-fig5-figsupp1-data1.zip › Figure 5 - figure supplement 1 Source Data/22.12.17_04.44.10 Saturation Concentration R42E Protein re Annotated.TIF]

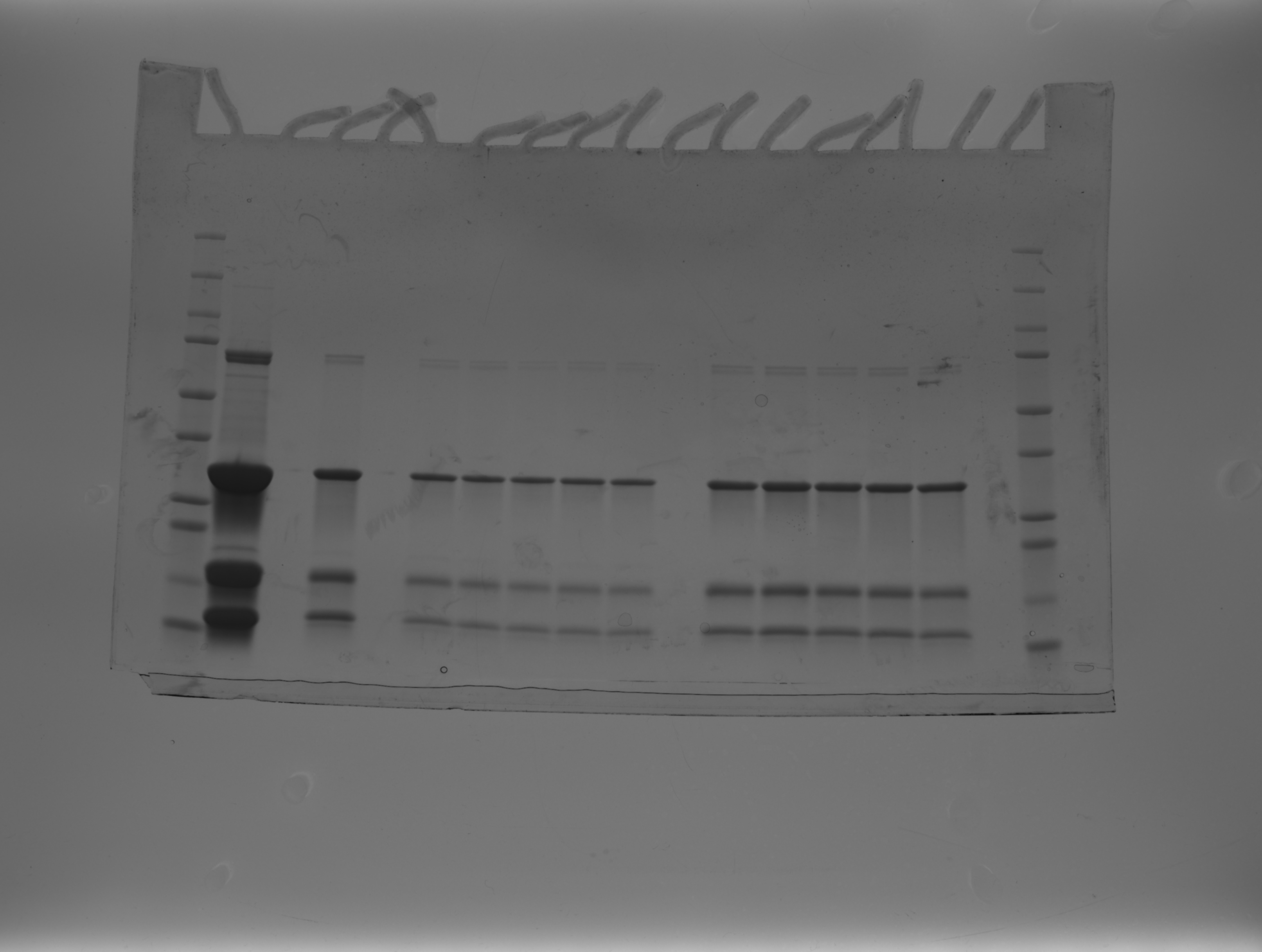

Supplement: Figure 5—figure supplement 1—source data 1. [file elife-92709-fig5-figsupp1-data1.zip › Figure 5 - figure supplement 1 Source Data/22.12.17_04.44.10 Saturation Concentration R42E Protein re.tif]

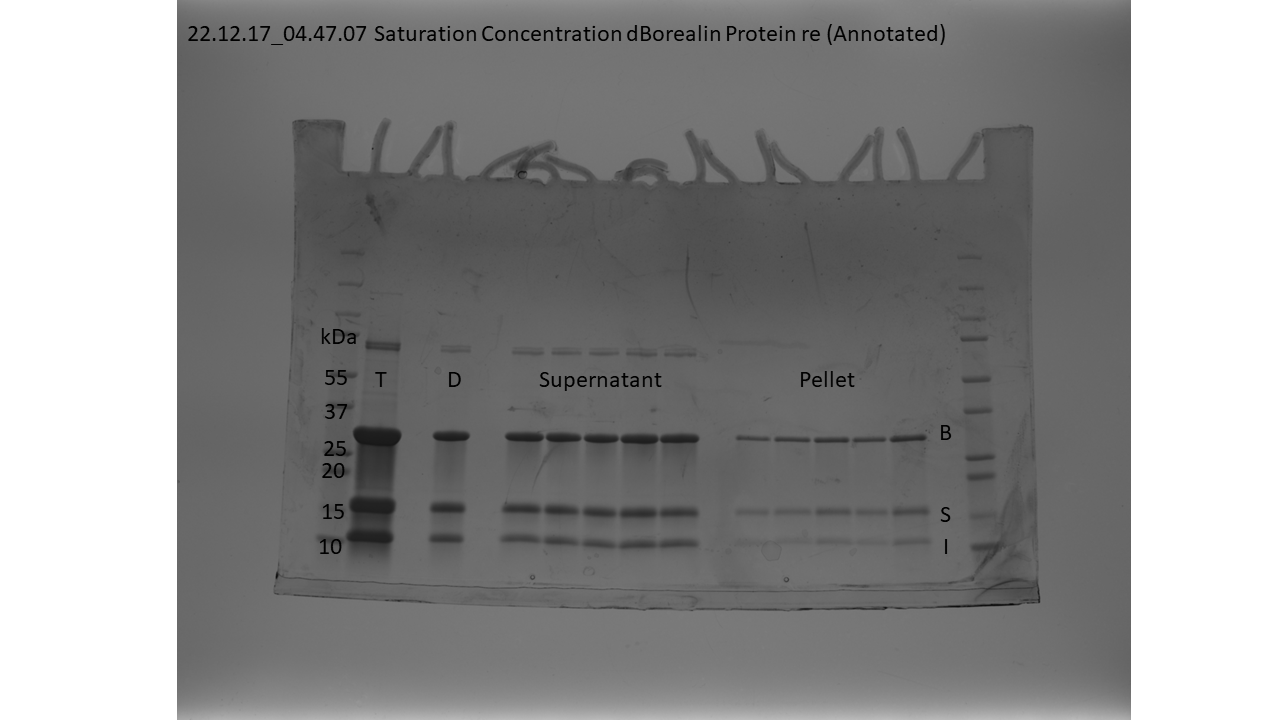

Supplement: Figure 5—figure supplement 1—source data 1. [file elife-92709-fig5-figsupp1-data1.zip › Figure 5 - figure supplement 1 Source Data/22.12.17_04.47.07 Saturation Concentration dBorealin Protein re Annotated.TIF]

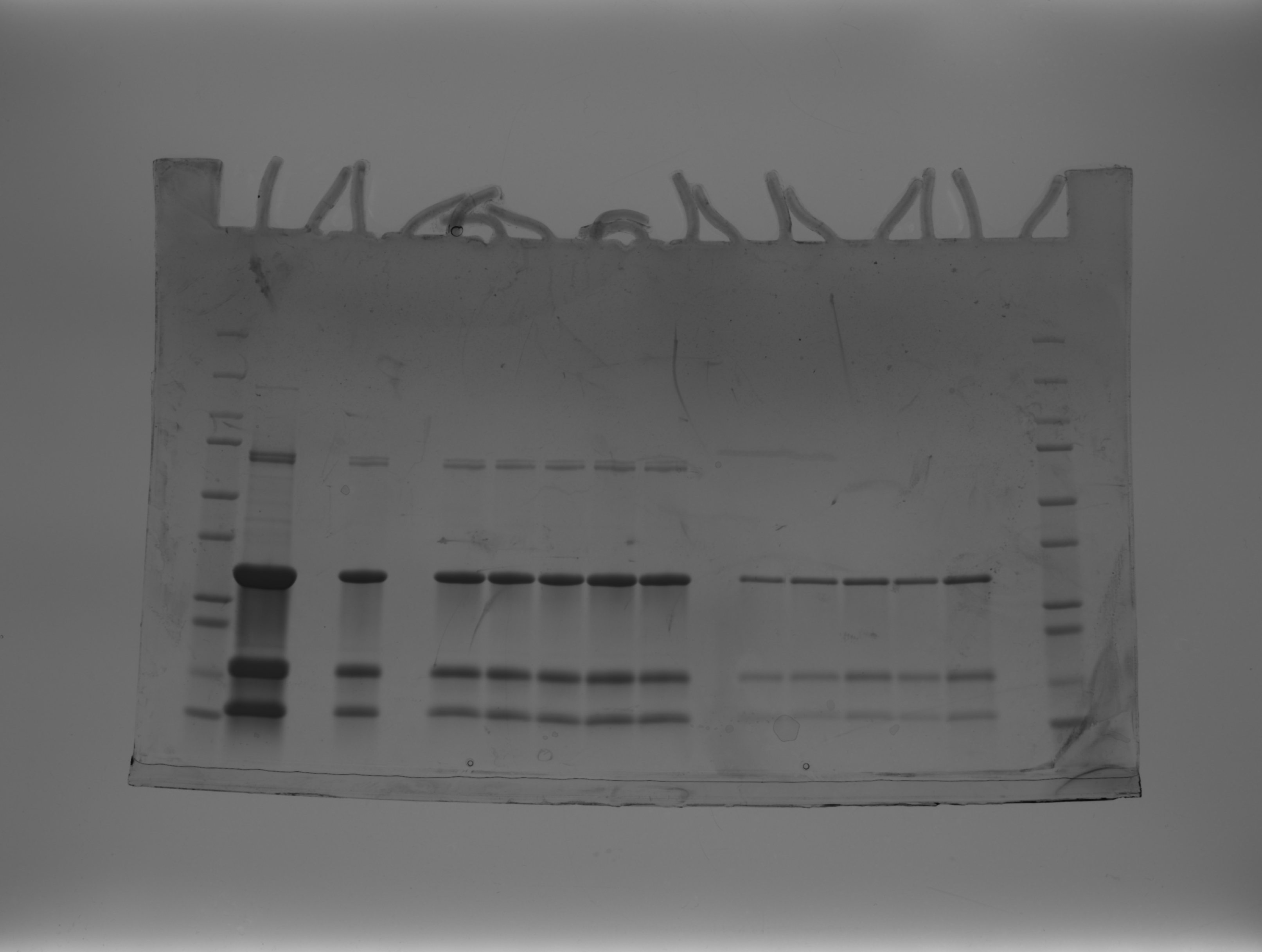

Supplement: Figure 5—figure supplement 1—source data 1. [file elife-92709-fig5-figsupp1-data1.zip › Figure 5 - figure supplement 1 Source Data/22.12.17_04.47.07 Saturation Concentration dBorealin Protein re.tif]

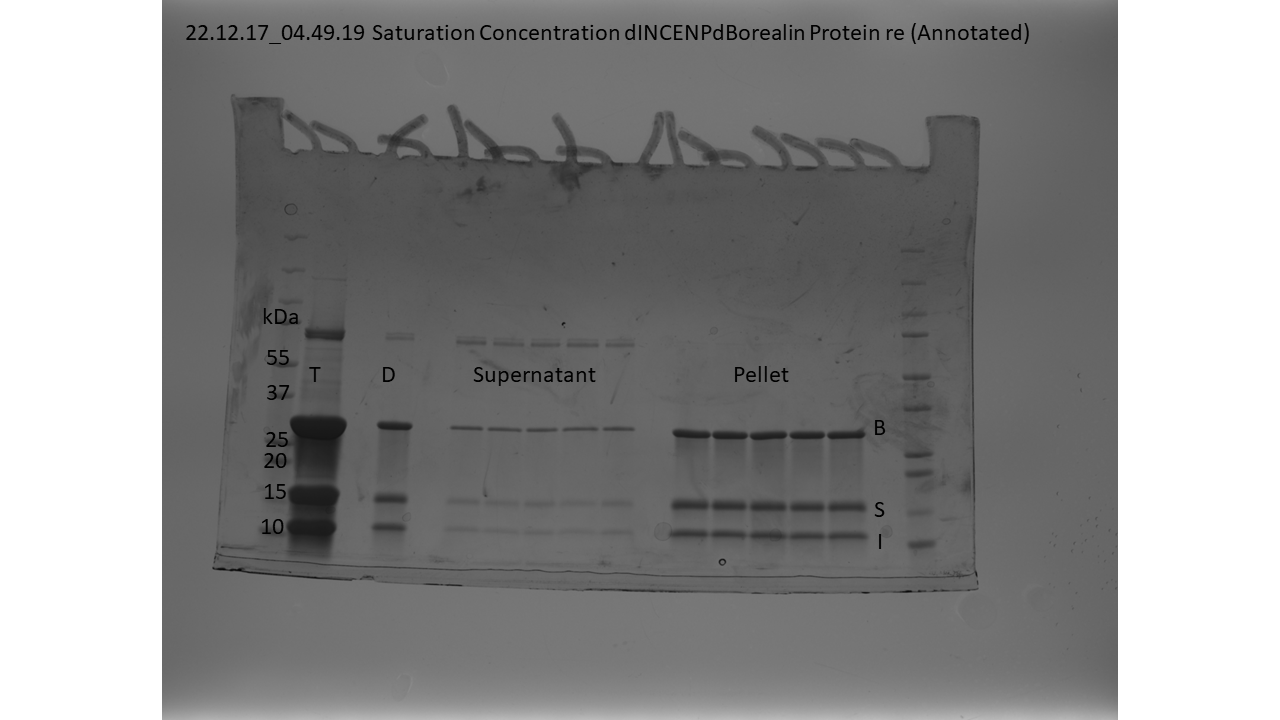

Supplement: Figure 5—figure supplement 1—source data 1. [file elife-92709-fig5-figsupp1-data1.zip › Figure 5 - figure supplement 1 Source Data/22.12.17_04.49.19 Saturation Concentration dINCENPdBorealin Protein re Annotated.TIF]

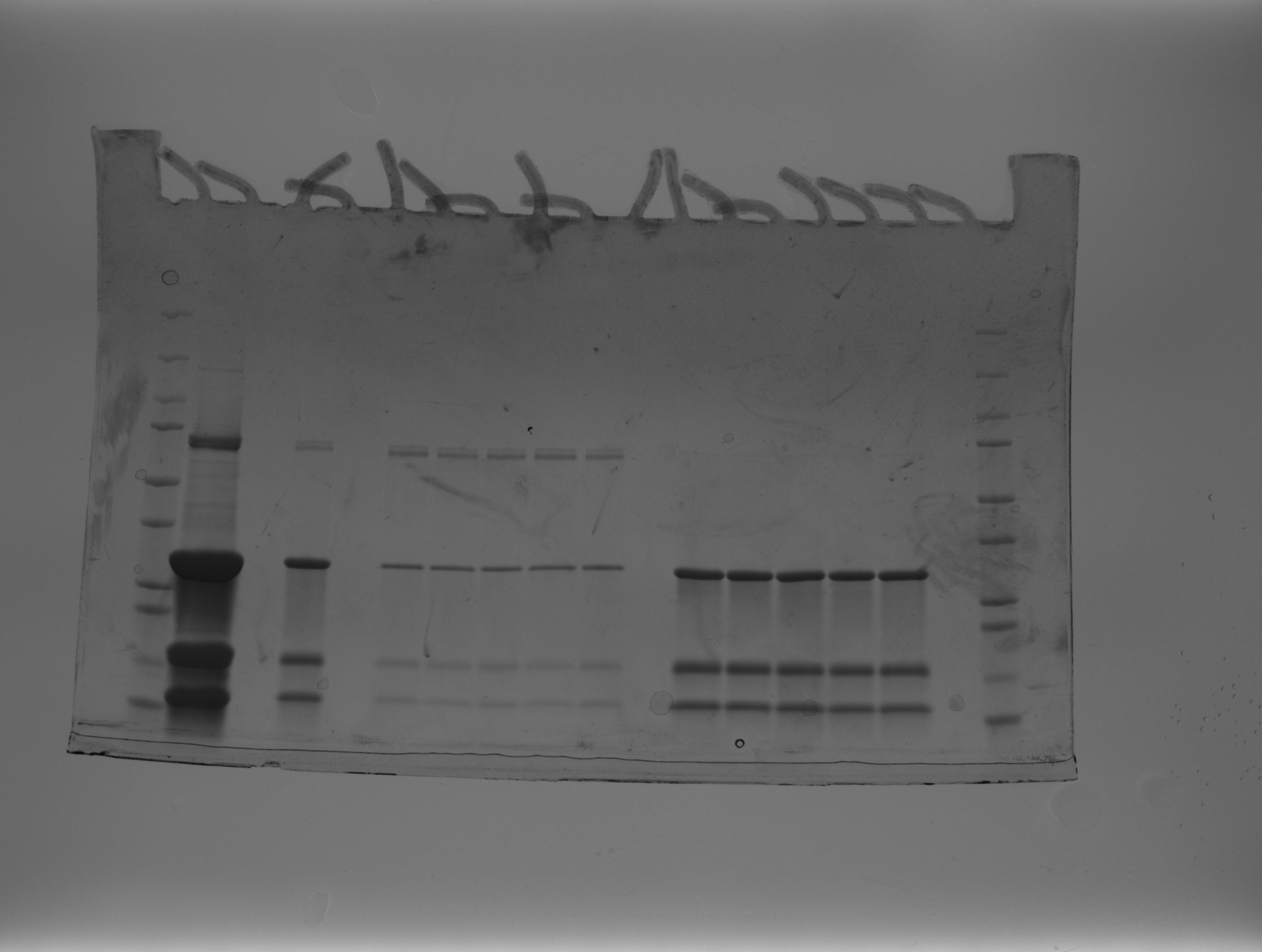

Supplement: Figure 5—figure supplement 1—source data 1. [file elife-92709-fig5-figsupp1-data1.zip › Figure 5 - figure supplement 1 Source Data/22.12.17_04.49.19 Saturation Concentration dINCENPdBorealin Protein re.tif]

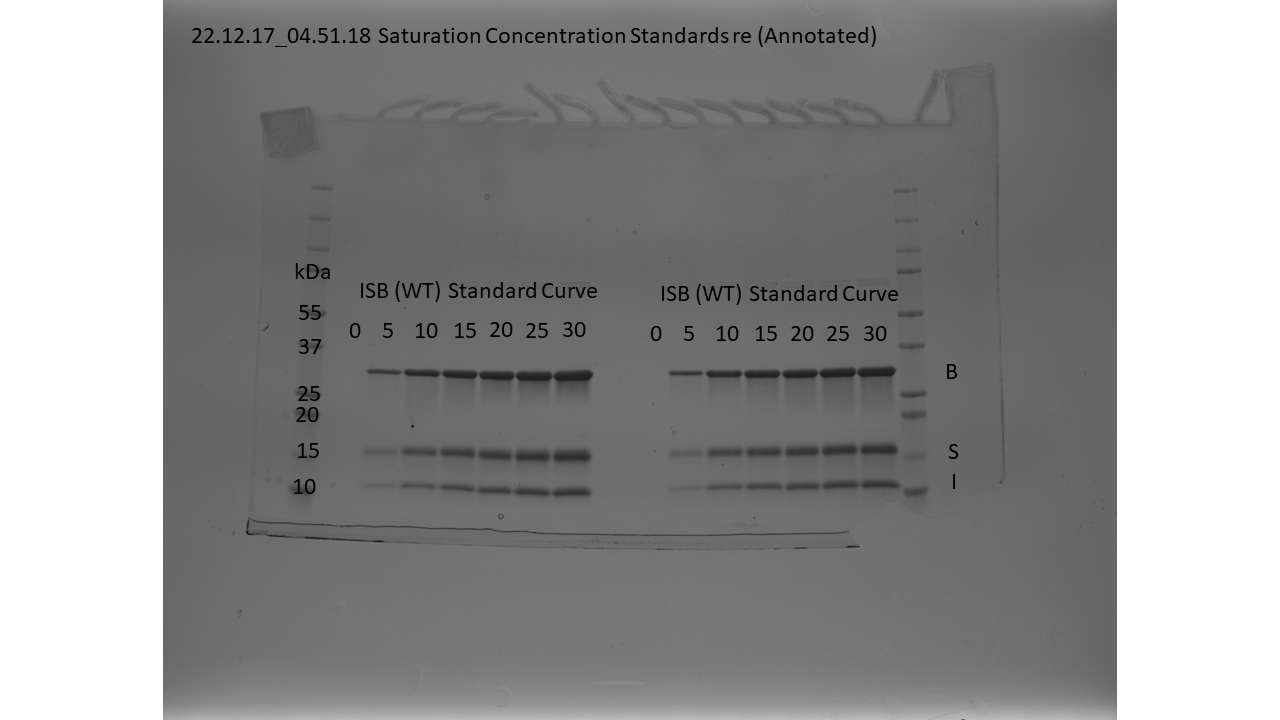

Supplement: Figure 5—figure supplement 1—source data 1. [file elife-92709-fig5-figsupp1-data1.zip › Figure 5 - figure supplement 1 Source Data/22.12.17_04.51.18 Saturation Concentration Standards re Annotated.TIF]

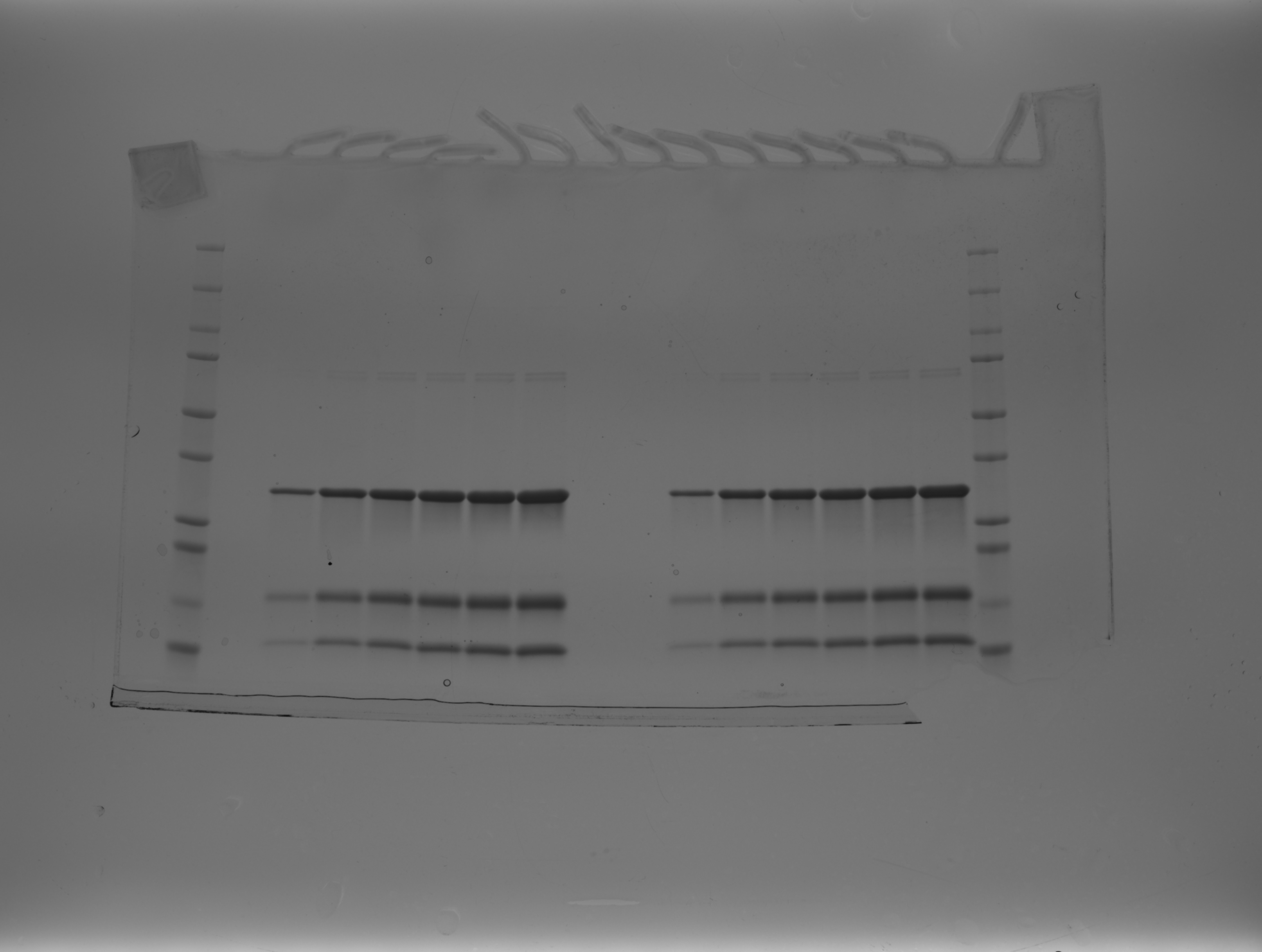

Supplement: Figure 5—figure supplement 1—source data 1. [file elife-92709-fig5-figsupp1-data1.zip › Figure 5 - figure supplement 1 Source Data/22.12.17_04.51.18 Saturation Concentration Standards re.tif]

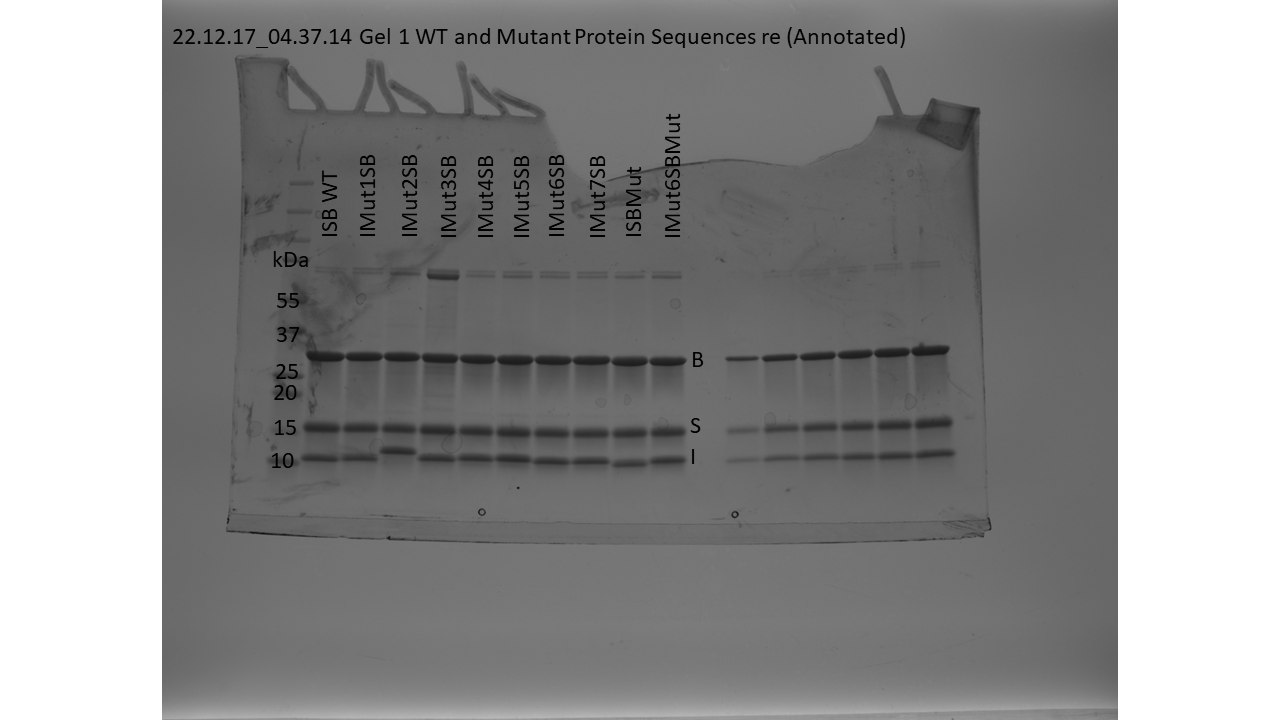

Supplement: Figure 5—figure supplement 2—source data 1. [file elife-92709-fig5-figsupp2-data1.zip › Figure 5 - figure supplement 2/22.12.17_04.37.14 Gel 1 WT and Mutant Protein Sequences re Annotated.TIF]
