# Supplementary material for: Structural basis for the phase separation of the chromosome passenger complex
Source: eLife. 2024 Mar 8;13:e92709. doi: 10.7554/eLife.92709 (PMC10977965; doi:10.7554/eLife.92709)
Supplement: Supplementary file 2. [file elife-92709-supp2.docx]

**Appendix**

**Gene Block Sequences**

**I_Mut1_SB G Block**: 3’ - CAG CAG CCA TCA TCA TCA TCA TCA CAG CAG CGG CCT GGT GCC GCG CGG CAG CCA TAT GGG GAC GAC GGC CCC AGG GCC CAT TCA CCT GCT GGA GCT ATG TGA CCA GAA GCT CAT GGA GTT TCT CTG CAA CAT GGA TAA TAA GGA CTT GGT GTG GCT TGC TGC CAT CCA AGC CGC AGC CGC TCG CAT GTT CAC CAG AGA ATT CAG CAA AGA GCC AGA GCT GAT GCC CAA ATG AGA TCC GAA TTC GAG CTC TAA TTT TG - 5’

**I_Mut2_SB G Block**: 3’ - CAG CAG CCA TCA TCA TCA TCA TCA CAG CAG CGG CCT GGT GCC GCG CGG CAG CCA TAT GGG GAC GAC GGC CCC AGG GCC CAT TCA CCT GCT GGA GCT ATG TGA CCA GAA GCT CAT GGA GTT TCT CTG CAA CAT GGA TAA TAA GGA CTT GGT GTG GCT TCG TCG AAT CCA ACG TCG AGC CCG TCG CAT GTT CAC CAG AGA ATT CAG CAA AGA GCC AGA GCT GAT GCC CAA ATG AGA TCC GAA TTC GAG CTC TAA TTT TG - 5’

**I_Mut3_SB G Block**: 3’ - CAG CAG CCA TCA TCA TCA TCA TCA CAG CAG CGG CCT GGT GCC GCG CGG CAG CCA TAT GGG GAC GAC GGC CCC AGG GCC CAT TCA CCT GCT GGA GCT ATG TGA CCA GAA GCT CAT GGA GTT TCT CTG CAA CAT GGA TAA TAA GGA CTT GGT GTG GCT TGA GCG TAT CCA AGA GCG AGC CGA GCG CAT GTT CAC CAG AGA ATT CAG CAA AGA GCC AGA GCT GAT GCC CAA ATG AGA TCC GAA TTC GAG CTC TAA TTT TG - 5’

**I_Mut4_SB G Block**: 3’ - CAG CAG CCA TCA TCA TCA TCA TCA CAG CAG CGG CCT GGT GCC GCG CGG CAG CCA TAT GGG GAC GAC GGC CCC AGG GCC CAT TCA CCT GCT

GGA GCT ATG TGA CCA GAA GCT CAT GGA GTT TCT CTG CAA CAT GGA TAA TAA GGA CTT GGT GTG GCT TGA GCG TAT CCA AGA GCG AGC CCG ACG CAT GTT CAC CAG AGA ATT CAG CAA AGA GCC AGA GCT GAT GCC CAA ATG AGA TCC GAA TTC GAG CTC TAA TTT TG - 5’

**I_Mut5_SB G Block**: 3’ - CAG CAG CCA TCA TCA TCA TCA TCA CAG CAG CGG CCT GGT GCC GCG CGG CAG CCA TAT GGG GAC GAC GGC CCC AGG GCC CAT TCA CCT GCT

GGA GCT ATG TGA CCA GAA GCT CAT GGA GTT TCT CTG CAA CAT GCG TAA TAA GGA CTT GGT GTG GCT TGA GCG TAT CCA AGA GCG AGC CCG ACG CAT GTT CAC CAG AGA ATT CAG CAA AGA GCC AGA GCT GAT GCC CAA ATG AGA TCC GAA TTC GAG CTC TAA TTT TG - 5’

**ISB_Mut_ G Block**: 3’ - CAG CAG CCA TCA TCA TCA TCA TCA CAG CAG CGG CCT GGT GCC GCG CGG CAG CCA TAT GGG GAC GAC GGC CCC AGG GCC CAT TCA CCT GCT

GGA GCT ATG TGA CCA GAA GCT CAT GGA GTT TCT CTG CAA CAT GGA TAA TAA GGA CTT GGT GTG GCT TGA GGA AAT CCA AGA GGA GGC CGA GCG CAT GTT CAC CAG AGA ATT CAG CAA AGA GCC AGA GCT GAT GCC CAA ATG AGA TCC GAA TTC GAG CTC TAA TTT TGT TTA ACT TTA AGA AGG AGA TAT ACC ATG GCT CCT AGG AAG GGC AGT AGT CGG GTG GCC AAG ACC AAC TCC TTA CGG AGG CGG AAG CTC GCC TCC TTT CTG GAG GAC TTC GAC CGT GAA GTG GAA ATA CGA ATC GAG CAA ATT GAG TCA GAC AGG CAG AAC CTC CTC AAG GAG GTG GAT AAC CTC TAC AAC ATC GAG ATC CTG CGG CTC CCC GAG GCT CTG CGC GAG ATG AAC TGG CTT GAC TAC TTC GCC CTT GGA GGA AAC AAA CAG GCC CTG GAA GAG GCG GCA ACA GCT GAC CTG GAT ATC ACC GAA ATA AAC AAA CTA ACA GCA GAA GCT ATT CAG ACA CCC CTG AAA TCT GCC AAA ACA CGA AAG GTA ATA CAG GTA GAT GAA ATG ATA GTG GAA GAG GAA GAA GAA GAA GAA AAT GAA CGT AAG AAT CTT CAA ACT GCA AGA GTC AAA AGG TGT CCT CCA

TCC AAG AAG AGA ACT CAG TCC ATG CAA GGA AAA GGA AAA GGG AAA AGG TCA AGC CGT GCT AAC ACT GTT ACC CCA GCC GTG GGC CGA TTG GAG GTG TCC ATG GTC AAA CCA ACT CCA GGC CTG ACA CCC AGG TTT GAC TCA AGG GTC TTC AAG ACC CCT GGC CTG CGT ACT CCA GCA GCA GGA GAG CGG ATT TAC AAC ATC TCA GGG AAT GGC AGC CCT CTT GCT GAC AGC AAA GAG ATC TTC CTC ACT GTG CCA GTG GGC GGC GGA GAG AGC CTG CGA TTA TTG GCC AGT GAC TTG CAG AGG CAC AGT ATT GCC CAG CTG GAT CCA GAG GCC TTG GGA AAC ATT AAG AAG CTC TCC AAC CGT CTC GCC CAA ATC TGC A - 5’

**I_Mut6_SB_Mut_ G Block**: 3’ - CAG CAG CCA TCA TCA TCA TCA TCA CAG CAG CGG CCT GGT GCC GCG CGG CAG CCA TAT GGG GAC GAC GGC CCC AGG GCC CAT TCA CCT GCT

GGA GCT ATG TGA CCA GAA GCT CAT GGA GTT TCT CTG CAA CAT GCG TAA TAA GGA CTT GGT GTG GCT TGA GCG TAT CCA AGA GGA GGC CCG ACG CAT GTT CAC CAG AGA ATT CAG CAA AGA GCC AGA GCT GAT GCC CAA ATG AGA TCC GAA TTC GAG CTC TAA TTT TGT TTA ACT TTA AGA AGG AGA TAT ACC ATG GCT CCT AGG AAG GGC AGT AGT CGG GTG GCC AAG ACC AAC TCC TTA CGG AGG CGG AAG CTC GCC TCC TTT CTG GAG GAC TTC GAC CGT GAA GTG GAA ATA CGA ATC GAG CAA ATT GAG TCA GAC AGG CAG AAC CTC CTC AAG GAG GTG GAT AAC CTC TAC AAC ATC GAG ATC CTG CGG CTC CCC GAG GCT CTG CGC GAG ATG AAC TGG CTT GAC TAC TTC GCC CTT GGA GGA AAC AAA CAG GCC CTG GAA GAG GCG GCA ACA GCT GAC CTG GAT ATC ACC GAA ATA AAC AAA CTA ACA GCA GAA GCT ATT CAG ACA CCC CTG AAA TCT GCC AAA ACA CGA AAG GTA ATA CAG GTA GAT GAA ATG ATA GTG GAA GAG GAA GAA GAA GAA GAA AAT GAA CGT AAG AAT CTT CAA ACT GCA AGA GTC AAA AGG TGT CCT CCA

TCC AAG AAG AGA ACT CAG TCC ATG CAA GGA AAA GGA AAA GGG AAA AGG TCA AGC CGT GCT AAC ACT GTT ACC CCA GCC GTG GGC CGA TTG GAG GTG TCC ATG GTC AAA CCA ACT CCA GGC CTG ACA CCC AGG TTT GAC TCA AGG GTC TTC AAG ACC CCT GGC CTG CGT ACT CCA GCA GCA GGA GAG CGG ATT TAC AAC ATC TCA GGG AAT GGC AGC CCT CTT GCT GAC AGC AAA GAG ATC TTC CTC ACT GTG CCA GTG GGC GGC GGA GAG AGC CTG CGA TTA TTG GCC AGT GAC TTG CAG AGG CAC AGT ATT GCC CAG CTG GAT CCA GAG GCC TTG GGA AAC ATT AAG AAG CTC TCC AAC CGT CTC GCC CAA ATC TGC A - 5’
